# Tumor microenvironment features decipher the outperformance of neoadjuvant immunochemotherapy over chemotherapy in resectable non-small cell lung cancer

**DOI:** 10.3389/fimmu.2022.984666

**Published:** 2022-10-06

**Authors:** Wenhan Cai, Miao Jing, Yajun Gu, Ting Bei, Xiaochen Zhao, Shiqing Chen, Jiaxin Wen, Jie Gao, Chongchong Wu, Zhiqiang Xue

**Affiliations:** ^1^ Department of Thoracic Surgery, the First Medical Center of Chinese PLA General Hospital, Beijing, China; ^2^ Department of Medical Affairs, 3D Medicines Inc., Shanghai, China; ^3^ Department of Pathology, the First Medical Center of Chinese PLA General Hospital, Beijing, China; ^4^ Department of Diagnostic Radiology, the First Medical Center of Chinese PLA General Hospital, Beijing, China

**Keywords:** non-small cell lung cancer, PD-(L) 1 blockade, tumor immune microenvironment, neoadjuvant therapy, immunochemotherapy

## Abstract

This study evaluated the efficacy of neoadjuvant immunochemotherapy (Io+Chemo) versus chemotherapy alone (Chemo) in resectable non–small cell lung cancer (NSCLC) in a real-world setting. The association of tumor immune microenvironment (TIME) with pathologic response to different neoadjuvant therapies was also explored.Stage I−III NSCLC patients who received Io+Chemo or Chemo alone followed by surgery were included in the study. Tumor tissues collected during surgery were subjected to TIME evaluation using multiplex immunohistochemistry to measure immune cell subsets, including T cells, B cells, NK cells, and macrophages. Fifty-five patients were included, including 24 treated with neoadjuvant Io+Chemo and 31 with Chemo alone. Io+Chemo induced significantly higher major pathologic response (MPR) (75.0% vs. 38.7%, *P* = 0.0133) and numerically better pathologic complete response (pCR) (33.3% vs. 12.9%, *P* = 0.1013) than Chemo. Compared with tumors with Chemo, tumors with Io+Chemo demonstrated a significantly higher ratio of M1 macrophage density in the tumor to that in the stroma (*P* = 0.0446), more abundant CD8^+^ cells in the stroma (*P* = 0.0335), and fewer PD-L1^+^CD68^+^ cells in both tumor and stroma. pCR/MPR patients displayed significantly higher density of CD3^+^, CD3^+^CD4^+^, CD20^+^, CD56 bright cell subsets and more tertiary lymphoid structures and significantly lower density of PD-L1^+^CD68^+^ and CD3^+^CD4^+^Foxp3^+^cells in the tumor or stroma. This study favored neoadjuvant Io+Chemo over Chemo and revealed the TIME features underlying the outperformance of Io+Chemo over Chemo.

## Introduction

Immunotherapies targeting cytotoxic T lymphocyte-associated protein 4 (CTLA4) and the axis of programmed death 1 (PD-1)/programmed death ligand-1 (PD-L1) have ushered the modern era of oncology. Following the approval of pembrolizumab as the frontline treatment for advanced and metastatic non-small cell lung cancers (NSCLC) patients who are PD-L1 positive, neoadjuvant use of anti-PD-L1/PD-1 antibody has been exploited (1). Increasing trials are currently underway to evaluate the preoperative utility of anti-PD-L1/PD-1 antibody in multiple malignancies, including lung cancer. CheckMate 159 (NCT02259621), a phase II trial, reported a major pathologic response (MPR) rate of 45% in stage I−III NSCLC with nivolumab (2). That rate from other studies of anti-PD-L1/PD-1 antibody decreased, ranging from 13.8% to 40.0% (3–8). More recently, the NADIM trial, which examined the combination of nivolumab with chemotherapy, has reported superior pathologic complete response (pCR) and MPR rates of 82.9% and 63.4%, respectively, and 36-month progression-free survival (PFS) and overall survival (OS) of 81.1% and 91.0%, respectively, among patients with stage IIIA NSCLC, showing great promise of PD-(L)1 blockade plus chemotherapy in shifting the paradigm of NSCLC (9, 10). Similarly, CheckMate 816 showed that neoadjuvant nivolumab plus chemotherapy increased MPR and pCR rate to 36.9% and 24.0%, respectively, in stage IB-IIIA NSCLC, and other trials (clinical trial NO. NCT02572843, NCT02716038, NCT04304248) released remarkably consistent MPR rate running the gamut between ~62% and ~67% and favorable pCR rate as well (11–14).

As a newcomer of “common dominator” for cancer therapy, immunotherapy of PD-(L)1 blockade exerts a distinct mechanism in comparison with chemotherapy. Whereas neoadjuvant chemotherapy aims to preoperatively “debulk” tumors to resectable ones, neoadjuvant PD-(L)1 blockades, termed normalization cancer immunotherapy, exploit strategy based on immune evasion mechanisms to restore antitumor immunity to defend tumor antigens. Anti-PD-(L)1 recovers the functional tumor-specific cytotoxic T cells in the tumor immune microenvironment (TIME). Moreover, neoadjuvant PD-(L)1 blockade leverages the high levels of tumor antigen in the primary tumor to enhance T cell priming (15). At present, extensive studies are unmet to better understand the mechanism actions for these two distinct therapeutic treatments. Particularly, the mechanisms underlying the outperformance of PD-(L)1 blockade plus chemotherapy were poorly studied. The co-effects of this combination on immune response and TIME could be illuminated by analyzing tumor specimens obtained after neoadjuvant treatment, which offered a rich source for in-depth interrogations. Findings from that studies may uncover pathways, mechanisms, and biomolecules that could be co-targeted in new treatment combinations to increase the efficacy of anti–PD-(L)1 drugs (15).

Except for CheckMate 816, few studies evaluated PD-(L) 1 blockade plus chemotherapy and chemotherapy alone in a head-to-head manner. This study investigates the treatment response to neoadjuvant treatment with Io+Chemo in comparison with Chemo alone in a real-world cohort of patients with resectable NSCLC. The associations of post-NAT TIME with treatment and treatment response were also explored, attempting to elucidate the mechanism underlying the effects of neoadjuvant immunotherapy plus chemotherapy.

## Materials and methods

### Participants and study design

**Figure 1:**
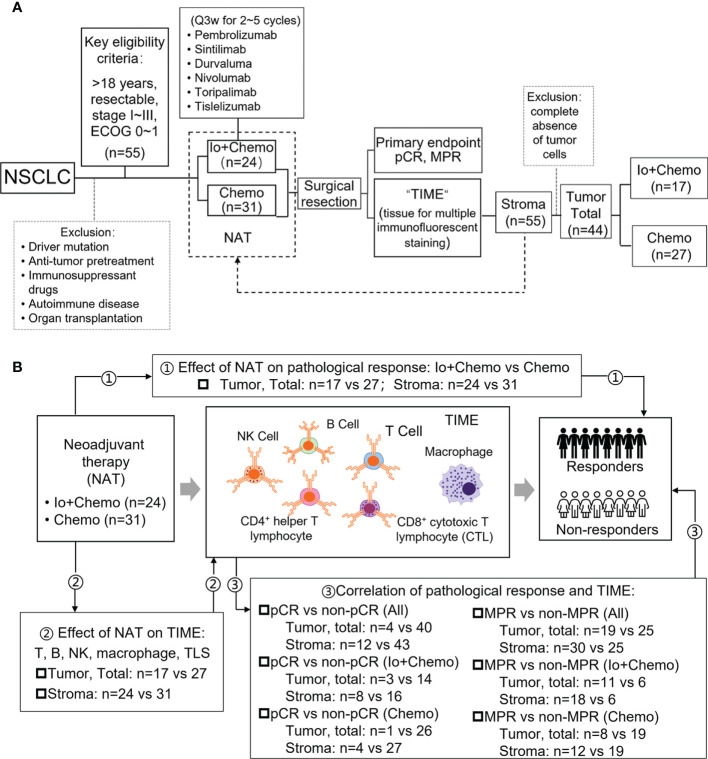
Study design examining effects of neoadjuvant therapies on resectable NSCLC patients. **(A)** Study flow chart depicting the study protocol. **(B)** The endpoints explored and sample details in each analyses. NSCLC, non-small cell lung cancer; TIME, tumor immune microenvironment; NAT, neoadjuvant therapy; pCR, pathological complete response; MPR, major pathological response; Io+Chemo, immunochemotherapy; Chemo, chemotherapy; CTL, cytotoxic T lymphocytes. Figure was created with Motifolio Toolkit (Motifolio Inc, Ellicott City, USA). *P <0.05; ns, no statistical significance.

NSCLC patients who received neoadjuvant immunotherapy combined with chemotherapy (Io+Chemo) or chemotherapy alone (Chemo), followed by surgery between October 5, 2018 and June 30, 2021 at the First Medical Center of Chinese PLA General Hospital were retrospectively included if they were aged over 18 years and had resectable stage I−III NSCLC, at least one radiologically measurable target lesion, and an Eastern Cooperative Oncology Group (ECOG) performance status (PS) of 0~1. Patients were excluded for having driver mutations (*EGFR* 19 deletion/L858R and *ALK* fusion), anti-tumor pretreatment, previous exposure to immunosuppressive drugs, autoimmune disease, and organ transplantation. All surgical specimens were subjected to pathologic response and TIME evaluation. This study aimed to investigate the effects of neoadjuvant Io+Chemo and Chemo on NSCLC patients and TIME. The association of post-NAT TIME with pathologic response was also explored (Fig. 1). The research protocol, standard operating procedure (SOP) of data collection, and case report form (CRF) were prospectively designed before the beginning of the study to guarantee the data quality. All procedures performed involving human participants were conducted in accordance with Declaration of Helsinki (as revised in 2013). This study was approved by the ethics committee of the First Medical Center of Chinese PLA General Hospital, and written informed consent was obtained from each patient.

### Assessment

Hematoxylin and eosin (H&E) staining was performed on the surgical resection to access pathologic responses to neoadjuvant therapy. An MPR was defined as having less than 10% residual viable tumor cells, and a pCR referred to no residual tumor cells. Computed tomography (CT) scans were conducted before and after neoadjuvant therapy to access radiologic responses of primary tumors.

### Multiplex immunofluorescence staining

Surgical tissue specimens were subjected to the examination of the TIME, which was performed as previously described by 3D Medicines, Inc., a College of American Pathologists (CAP)-accredited and Clinical Laboratory Improvement Amendments (CLIA)-certified laboratory (16). The Akoya OPAL Polaris 7-Color Automation IHC kit (NEL871001KT) was applied to conduct multiplex immunofluorescence (mIF) staining following manufacturer’s instructions. Primary antibodies targeting CD163 (Abcam, ab182422, 1:500), CD68 (Abcam, ab213363, 1:1000), PD-1 (CST, D4W2J, 86163S, 1:200), PD-L1 (CST, E1L3N, 13684S, 1:400), CD3 (Dako, A0452, 1:1), CD4 (Abcam, ab133616, 1:100), CD8 (Abcam, ab178089, 1:200), CD56 (Abcam, ab75813, 1:1000), CD20 (Dako, L26, IR604, 1:1), Foxp3 (Abcam, ab20034, 1:100) and pan-CK (Abcam, ab7753, 1:100) or S100 (Abcam, ab52642, 1:200) were sequentially applied to FFPE tissue slides, followed by incubation with secondary antibodies and horseradish peroxidase and tyramide signal amplifying reagent. Nuclei acids were stained with DAPI. Multiplex stained slides were scanned using a Vectra Polaris Quantitative Pathology Imaging System (Akoya Biosciences), which was configured to capture fluorescent spectra at 20 nm wavelength intervals from 440 nm to 780 nm with a fixed exposure time and an absolute magnification of ×200. All scans for each slide were then superimposed to obtain a single image. Unstained and monoplex stained slide images were applied to extract tissue autofluorescence and the spectrum of each fluorophore, respectively. Fluorescence images were imported and analyzed using the AP-TIME image analysis software (3D Medicines Inc.) (17). Tumor parenchyma and stroma were differentiated according to CK staining. The CK positive area with DAPI staining was defined as tumor region, and the CK negative area with DAPI staining was considered as stroma region. The quantities of various cell subsets were expressed as the count number of positively stained cells per square millimeter (cells per mm^2^) and as the percentage of positively stained cells in all nucleated cells (%). Total density = (tumor cell counts + stroma cell counts)/(tumor area + stroma area). Total percentage = (tumor cell counts + stroma cell counts)/(tumor total cells + stroma total cells) ×100%. The density and percentage of immune cell subsets in tumor and stroma regions were figured out by detecting signal channel or multiple-channel, namely CD3^+^, CD3^+^CD4^+^, CD8^+^, Foxp3^+^, PD-1^+^CD8^+^, CD4^+^Foxp3^+^ (Treg), CD68^+^CD163^-^ (M1 macrophage), CD68^+^CD163^+^ (M2 macrophage), PD-L1^+^ CD68^+^, CD56 bright (NK cell), CD56 dim (NK cell). The co-occurrence of CD3^+^ T cells and CD20^+^ B cells indicates the formation of tertiary lymphoid structures (TLS).

### Statistical analysis

The statistical analyses were performed using the Graphpad Prism 9.2 software. Fisher’s exact test was used to analyze categorical variables (including NAT efficacy, age, sex, stage, pathology, smoking, and diabetes) between treatment groups. Comparisons between continuous variables with (i.e. BMI) normal distribution were performed using the unpaired t test, and the data with non-normal distribution (i.e. immune cell density) was analyzed by Mann−Whitney U test. *P* < 0.05 was considered statistically significant. The forest plots were built using ggplot2 package (R version 3.6.3). Logistic regression was used to investigate the association between baseline characteristics and pathologic response.

## Results

### Baseline characteristics

A total of 55 NSCLC patients who received Io+Chemo or Chemo alone before surgery and met the eligibility criteria were included in the study (**Figure 1** and **Table 1**), including 24 in the Io+Chemo group and 31 in the Chemo alone group. Baseline characteristics were balanced between the two treatment groups. The median age of the entire cohort was 61 years (range, 38−72 years). Most patients were male (51/55, 92.73%) and smokers (46/55, 83.64%). Half of the patients had a stage III disease, and lung squamous cell carcinomas (39/55, 70.91%) was the predominant pathologic type.

**Table 1:** Baseline characteristics of NSCLC patients with neoadjuvant therapy.

Characteristics				Io+Chemo vs. Chemo
	All (n = 55)	Io+Chemo (n = 24)	Chemo (n = 31)	P value
Age, years				0.558
Median (range) ≥65, n (%) <65, n (%)	61 (38~72) 17 (30.91%) 38 (69.09%)	58.5 (38~72) 6 (25.00%) 18 (75.00%)	62 (43~72) 11 (35.48%) 20 (64.52%)	
Sex, n (%)				1.000
Male Female	51 (92.73%) 4 (7.27%)	22 (91.67%) 2 (8.33%)	29 (93.55%) 2 (6.45%)	
Stage, n (%) before NAT				0.844
I II III	13 (23.64%) 12 (21.82%) 30 (54.55%)	5 (20.83%) 6 (25.00%) 13 (54.17%)	8 (25.81%) 6 (19.35%) 17 (54.84%)	
Pathology, n (%)				0.565
Sq Non-Sq	39 (70.91%) 16 (29.09%)	16 (66.67%) 8 (33.33%)	23 (74.19%) 8 (25.81%)	
Smoking, n (%)				0.716
Yes No	46 (83.64%) 9 (16.36%)	21 (87.50%) 3 (12.50%)	25 (80.65%) 6 (19.35%)	
Diabetes, n (%)				0.643
Yes No	5 (9.09%) 50 (90.91%)	3 (12.50%) 21 (87.50%)	2 (6.45%) 29 (93.55%)	
BMI, (kg/m^2^)				0.677
Mean ± SD	24.97 ± 3.01	25.17 ± 3.42	24.82 ± 2.71	

Sq, lung squamous cell carcinomas; NAT, neoadjuvant therapy.

### Addition of immunotherapy to chemotherapy increased the NAT efficacy

**Figure 2:**
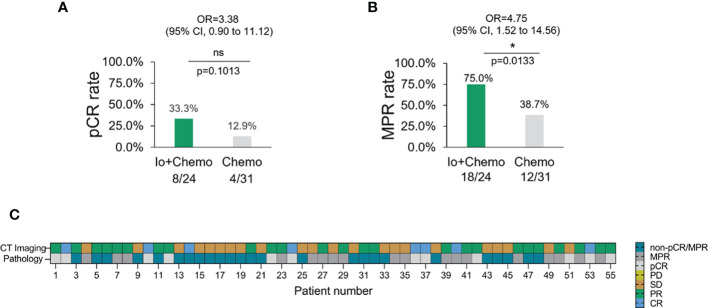
The pathologic response in NSCLC patients with different neoadjuvant therapy. **(A)** pCR rate among NSCLC patients with neoadjuvant Io+Chemo or Chemo alone therapy. **(B)** MPR rate among NSCLC patients with neoadjuvant Io+Chemo or Chemo alone therapy. **(C)** Concordance between pathologic and radiologic response. pCR, pathological complete response; MPR, major pathological response; Io+Chemo, immunochemotherapy; Chemo, chemotherapy. CT, computed tomography. *P < 0.05; ns, no statistical significance.

Pathologic response of primary tumor from each patient was evaluated for neoadjuvant efficacy. 12 patients achieved a pCR and thirty obtained an MPR. No association was found between pathologic response and baseline characteristics (**Supplementary Figure S1**). Patients who received Io+Chemo displayed significantly higher MPR rate (75.0% vs. 38.7%, *P* = 0.0133) and numerically increased pCR rate (33.3% vs 12.9%, *P* = 0.1013) than those with Chemo alone (**Figure 2**). The above data were comparable to the results from the trials, which evaluated the combination of chemotherapy and immunotherapy in resectable NSCLC patients (**Supplementary Figure S2**) (11–14).

### Distinct immune cell infiltration upon neoadjuvant immunochemotherapy and chemotherapy alone

**Figure 3:**
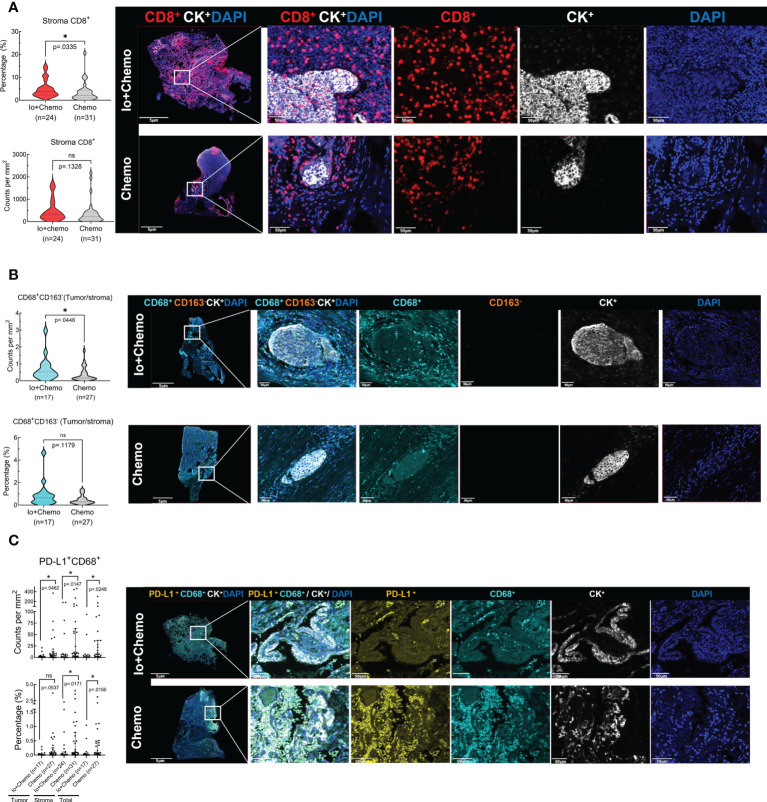
The immune cell biomarkers of tumor tissue samples from patients treated with neoadjuvant immunochemotherapy and chemotherapy alone. Multiplex immunofluorescence staining was performed for immune cell biomarkers, as denoted by different colors, in specimens of NSCLC patients treated with neoadjuvant therapy (surgical resection after NAT). The density and percentage of CD8^+^ **(A)**, CD68^+^CD163^-^ **(B)**, and PD-L1^+^CD68^+^ **(C)** immune cells in the tumor center or stroma were analyzed. Representative images showing the multiplex immunofluorescence staining for identifying the immune cell subsets in the tumor immune microenvironment. Io+Chemo, immunochemotherapy; Chemo, chemotherapy; *P <0.05; ns, no statistical significance.

Surgical tissue specimens were subjected to mIF to examine the TIME upon NAT. Of the 55 tissue samples, 11 were identified as tumor-free for a complete absence of tumor cells according to the results of CK and DAPI staining. Thus, immune cell infiltration was evaluated in all 55 cases of tumor stroma and in 44 cases of tumor. The density and percentage of immune cell subsets in TIME were quantified. The CD8^+^ cell was significantly more abundant in the stroma of the Io+Chemo group than that in the Chemo alone (*P* = 0.0335, **Figure 3A**). Compared with the Chemo group, the Io+Chemo group demonstrated a significantly higher M1 macrophage density (CD68^+^CD163^-^ cell subset) ratio in the tumor to that in the stroma (*P* = 0.0446; **Figure 3B**). A lower degree of infiltration of PD-L1^+^CD68^+^ cells was seen in both tumor and stroma in the Io+Chemo over in the Chemo (density: tumor, *P* = 0.0462, stroma, *P* = 0.0147, total, *P* = 0.0248; percentage: tumor, *P* = 0.0537, stroma, *P* =0.0171, total, *P* = 0.0156; **Figure 3C**). Such a decrease in the abundance of PD-L1^+^CD68^+^ cells could be explained by the fact that the PD-L1 on the surface of macrophages was thoroughly blocked by anti-PD-L1 antibodies upon immunotherapy. No difference was found in the infiltration of other immune cell subsets between the two NAT groups (**Tables S1**, **S2**).

### The association between pathologic response and TIME upon NAT

**Figure 4:**
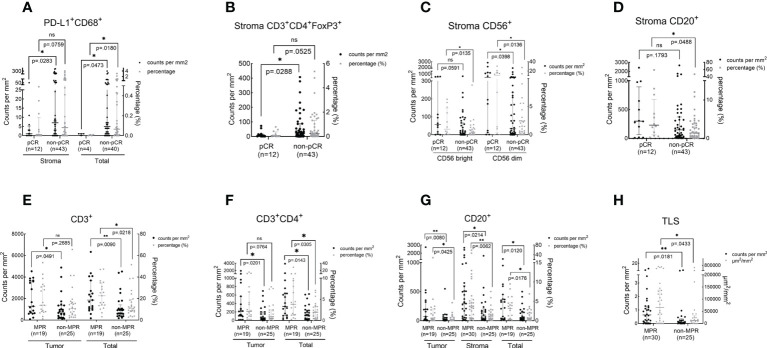
The association between immune cell infiltration in the TIME and pathologic response. The scatter plot was shown as median with interquartile range. pCR, pathological complete response; MPR, major pathological response; *P <0.05; *P <0.01; ns, no statistical significance. The density and percentage of PD-L1+CD68+ **(A)**, CD3+CD4+FoxP3+**(B)**, CD56+**(C)**, CD20+**(D, G)**, CD3+**(E)**, CD3+CD4+**(F)** immune cells and TLS **(H)** were statistical different in responders and non-responders. **p<0.01.

We sought to analyze whether pathologic responses were associated with TIME upon NAT and found that patients who achieved pCR showed a significantly lower infiltration of PD-L1^+^CD68^+^ (total, *P* = 0.018) and CD3^+^CD4^+^ Foxp3^+^ cells (stroma, *P* = 0.0288) and a higher density of CD56^+^ (stroma CD56 bright, *P* = 0.0135; stroma CD56 dim, *P* = 0.0136) and CD20^+^ cells (stroma, *P* = 0.0488) in the TIME over the non-pCR counterparts (**Figures 4A–D**). CD3^+^ (tumor, *P* = 0.0491; total, *P* = 0.0218), CD3^+^CD4^+^ (tumor, *P* = 0.0201; total, *P* = 0.0305), and CD20^+^ cells (tumor, *P* = 0.0425; stroma, *P* = 0.0214; total, *P* = 0.0176) and TLS (*P* = 0.0433) were more abundant in the TIME of MPR patients (**Figures 4E–H**) over that of the non-MPR patients. No difference was found in the infiltration of other immune cell subsets between the different responding groups (**Tables S3–S5**).

In patients who received Io+Chemo, no difference was found in immune cell infiltration between the responders and non-responders. A numerically higher density of TLS was observed in the TIME of MPR patients (**Figure S3A** and **Tables S6–S8**). While in the patients treated with Chemo, patients who achieved pCR were found to have a significantly lower density of Foxp3^+^ cells over the non-pCR patients (stroma, *P* = 0.038). MPR patients showed a significantly higher infiltration of CD3^+^ cells (Total, *P* = 0.0448), CD20^+^ cells (stroma, *P* = 0.0254), and TLS (*P*= 0.0063) (**Figures S3B−E** and **Tables S9–S11**).

## Discussion

**Figure 5:**
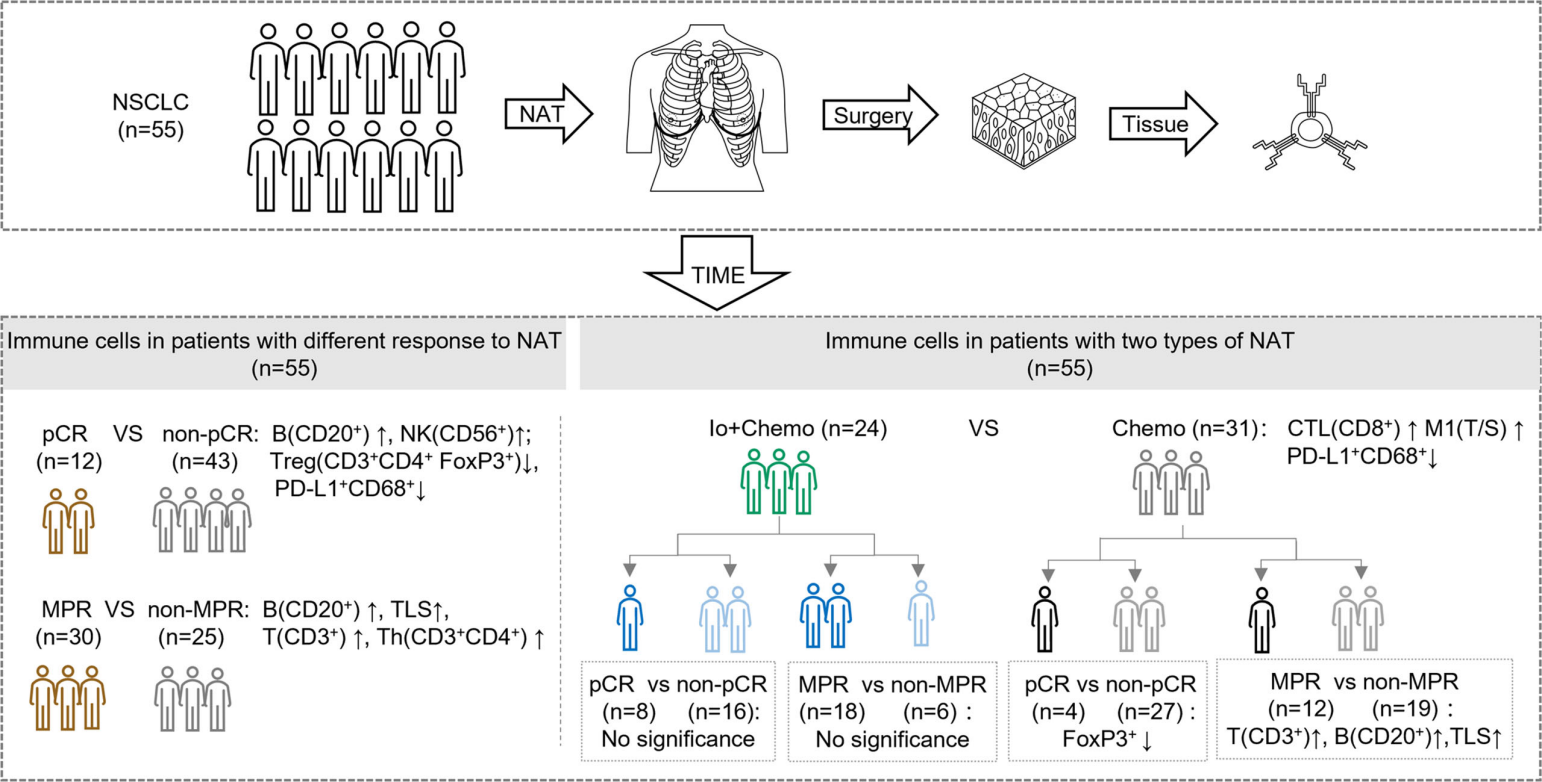
Summary of tumor microenvironment in patients with neoadjuvant therapy (NAT). TIME, tumor immune microenvironment; Io+Chemo, immunochemotherapy; Chemo, chemotherapy; pCR, pathological complete response; MPR, major pathological response. TLS, tertiary lymphoid structures; CTL, cytotoxic T lymphocytes; T/S, the ratio of tumor to stroma; M1, CD68^+^CD163^-^ macrophage. No significance means no difference at a given significance value (*P <*0.05). Figure was created with Motifolio Toolkit (Motifolio Inc, Ellicott City, USA).

In this real-world cohort of stage I-III resectable NSCLC patients, we report that the addition of PD-(L)1 blockade to chemotherapy was associated with an significantly increased MPR rate and a numerically higher pCR rate in comparison to chemotherapy alone (MPR, 75.0% vs. 38.7%; pCR, 33.3% vs. 12.9%), which favored PD-(L)1 blockade plus chemotherapy over chemotherapy alone. mIF analysis of surgical resection specimens revealed that compared with patients subjected to NAT of Chemo alone, patients treated with Io+Chemo showed more abundant CD8^+^ cells in tumor stroma and a higher ratio of M1 macrophage density in the tumor center to that in the tumor stroma, suggesting the potential mechanism underlying a better response to Io+Chemo than Chemo alone. Among the entire cohort, patients who obtained MPR or pCR displayed significantly increased infiltration of CD20^+^ B cells, CD3^+^ T cells, CD3^+^CD4^+^ T cells, CD56^+^ NK cells, TLS, and lower density of CD3^+^CD4^+^Foxp3^+^ nTreg cells and PD-L1^+^CD68^+^ cells compared with their non-MPR or non-pCR counterparts. In the Chemo alone group, increased infiltrations of CD20^+^ B cells, CD3^+^ T cells, and TLS were observed in MPR tumors over non-MPR ones, and a lower degree of infiltration of Foxp3+ cells was seen in the pCR tumors than that in the non-pCR tumors. In the Io+Chemo subgroup, no significant difference was found in the density of immune cell subsets between groups based upon response (**Figure 5**).

Most recently, CheckMate 816 has reported a significantly increased pathologic response induced by neoadjuvant nivolumab + chemotherapy over chemotherapy alone in stage IB to IIIA resectable NSCLC (11), which was slightly lower than that observed in our real-world cohort. Similarly, multiple single-arm trials released drastically increased MPR and pCR rates achieved from PD-(L)1 blockade plus chemotherapy (11–14). It is getting clear that the combinational strategy incorporating immune checkpoint inhibitors and chemotherapy is becoming the “primary actor” in the neoadjuvant NSCLC scenario. While cellular and molecular mechanism of PD-(L)1 blockade therapy has been studied, little is known about the mechanism underlying the outperformance of the combination of PD-(L)1 blockade with chemotherapy over chemotherapy alone. Our study evaluated the infiltration of immune cell subsets in the TIME utilizing the tumor tissue specimens collected after NAT (surgical specimen). A significantly higher degree of CD8^+^ T cell infiltration was observed in Io+Chemo than that in Chemo alone, suggesting PD-(L)1 blockade more robustly restored antitumor immunity by promoting cytotoxic T cell activation and proliferation. Consistently, Forde P et al. observed an increased number of T-cell clones in both the tumor and peripheral blood after preoperative treatment of nivolumab, and other research groups also reported similar evidence across multiple tumor types, including lung cancer, ovarian cancer, colorectal cancer, and esophageal squamous cell carcinoma (2, 8, 18, 19).

Moreover, compared with those with Chemo alone, tumors upon Io+Chemo showed a higher ratio of M1 macrophage density in the tumor center to that in the tumor stroma, making it rational to speculate that PD-(L)1 blockade improved the polarization of M1-TAMs and promoted the infiltration of M1-TAMs from tumor stroma to tumor center. This observation was consistent with previous reports that M1-TAMs may elevate antitumor immunity by producing immune-activating cytokines, rendering the patients responsive to immunotherapy (19, 20). Interestingly, we observed a decrease in the abundance of PD-L1^+^CD68^+^ macrophages in the Io+Chemo-treated tumor stroma over that of Chemo alone. The potential reasons that might give explanations for this phenomenon are the followings. First, PD-L1 that on the surface of microphages might be pre-blocked by anti-PD-L1 antibody (the immunotherapy regimen applied) before performing mIF. Second, we assumed that the immune-chemotherapy enhanced (or restored, if the PD-1/PD-L1 pathway is upregulated) antitumor immunity by altering the molecular characteristics of immune cell subsets to activate antitumor immune pathways, which involved the regulation of PD-L1 expression on macrophages. If the case was the second, it suggests that PD-(L)1 expression might not be the major hurdle for cancer patients who are less responsive to PD-(L)1 blockade. Perhaps the most novel look herein was that the combination of PD-(L)1 blockade with chemotherapy exerted similar effects on the TIME, such as increased infiltrations of CD8^+^ T cells and promoted polarization of M1 TAMs, as reported in studies investigating mono-immunotherapy of PD-(L)1 blockade. At least, chemotherapy, as a component of the combinatorial therapy regimen, might not have played a rogue role for efficacy.

Based on the evidence that both neoadjuvant immunotherapy and chemotherapy can induce immune responses fine-tuned by stimulation and inhibitory signals pathways (2, 21–24), we further examined the association between pathologic response and TIME regardless of the therapy strategy. In the entire cohort, patients who obtained MPR or pCR displayed significantly increased infiltration of CD20^+^ B cells, CD3^+^ T cells, CD3^+^CD4^+^ T cells, CD56^+^ NK cells, TLS, and decreased infiltration of CD3^+^CD4^+^Foxp3^+^ Treg cells and PD-L1^+^CD68^+^ cells. Thus, we envision that the tumors achieving pathologic response should display an enhanced antitumor immune response by regulating T lymphocytes and B lymphocytes through multiple immune pathways, either induced by chemotherapy or immunotherapy.

We further examined the association between pathologic response and TIME in treatment subgroups. In the Io+Chemo population, no difference was found in immune cell infiltration between the responders and non-responders, which might resulted from a small sample size. A numerically higher density of TLS was observed in the TIME of MPR patients. While in the patients who were treated with Chemo, MPR patients showed a significantly higher infiltration of TLS, CD3^+^ cells and CD20^+^ cells. Patients who achieved pCR were found to have a significantly lower density of FoxP3^+^ cells, which was consistent with previous reports that neoadjuvant chemotherapy increased cytotoxic T Cell, and B cell infiltration and decreased the density of Foxp3^+^ T cells (23) in the tumor of resectable NSCLC patients (21, 22).

This study was primarily limited by the small size and its retrospective design. Prospective studies with larger sample sizes are warranted to confirm the findings. Another limitation was that pre-surgery biopsy samples were not available, for which the exploration of the predictive value of pre-surgery TIME for efficacy and the comparison of TIME before and after NAT were not feasible.

## Conclusions

This real-world study favored neoadjuvant PD-(L)1 blockade plus chemotherapy over chemotherapy alone. We revealed for the first time that compared with chemo alone, Io+Chemo therapy was associated with increased infiltration of CD8^+^ T cells, and promoted polarization of M1 macrophages. Our findings provided new insights of understanding the mechanisms underlying the outperformance of Io+Chemo over Chemo alone.

## Data availability statement

The raw data supporting the conclusions of this article will be made available by the authors, without undue reservation.

## Ethics statement

This study was approved by the ethics committee of the First Medical Center of Chinese PLA General Hospital, and written informed consent was obtained from each patient. The patients/participants provided their written informed consent to participate in this study.

## Author contributions

WC: Acquisition of data, writing-review and editing. MJ: Methodology, writing-review and editing. YG: Data curation, formal analysis, writing-review and editing. TB: Writing–original draft, writing–review and editing. XZ: Conceptualization, resources, supervision, writing–review and editing. SC: Conceptualization, resources, supervision, writing–review and editing. JW: Resources, data curation, writing-review and editing. JG: Resources, data curation, writing-review and editing. CW: Resources, data curation, writing-review and editing. ZX: Conceptualization, resources, supervision, methodology, project administration, writing–review and editing. All authors contributed to the article and approved the submitted version.

## Funding

This work was supported by the National Natural Science Foundation of China (NO. 62076254).

## Acknowledgments

We would like to thank all coordinators at the First Medical Center of Chinese PLA General Hospital, China and 3D Medicines Inc., Shanghai, China for supporting this study.

## Conflict of interest

YG, TB, XZ and SC are employees of 3D Medicines Inc. The other authors declare no potential conflicts of interest.

## Publisher’s note

All claims expressed in this article are solely those of the authors and do not necessarily represent those of their affiliated organizations, or those of the publisher, the editors and the reviewers. Any product that may be evaluated in this article, or claim that may be made by its manufacturer, is not guaranteed or endorsed by the publisher.
