# Supplementary material for: Tumor microenvironment features decipher the outperformance of neoadjuvant immunochemotherapy over chemotherapy in resectable non-small cell lung cancer
Source: Front Immunol. 2022 Oct 6;13:984666. doi: 10.3389/fimmu.2022.984666 (PMC9582151; doi:10.3389/fimmu.2022.984666)
Supplement: Supplementary file 1 [file DataSheet_1.docx]

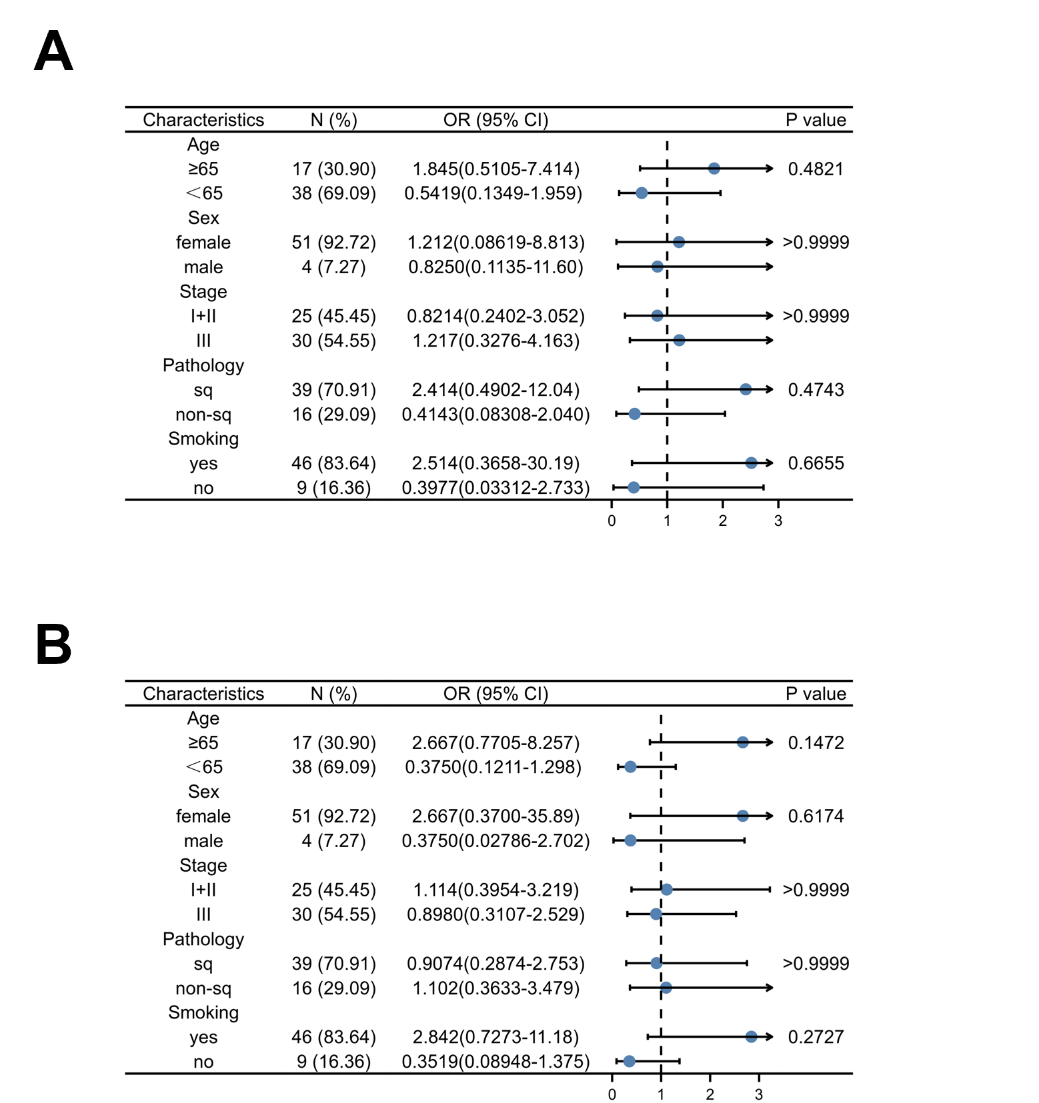


**Supplementary Figure 1 Association between baseline characteristics and pathologic response.** A. Association between baseline characteristics and complete pathologic response. B. Association between baseline characteristics and major pathologic response. OR, odds ratio.


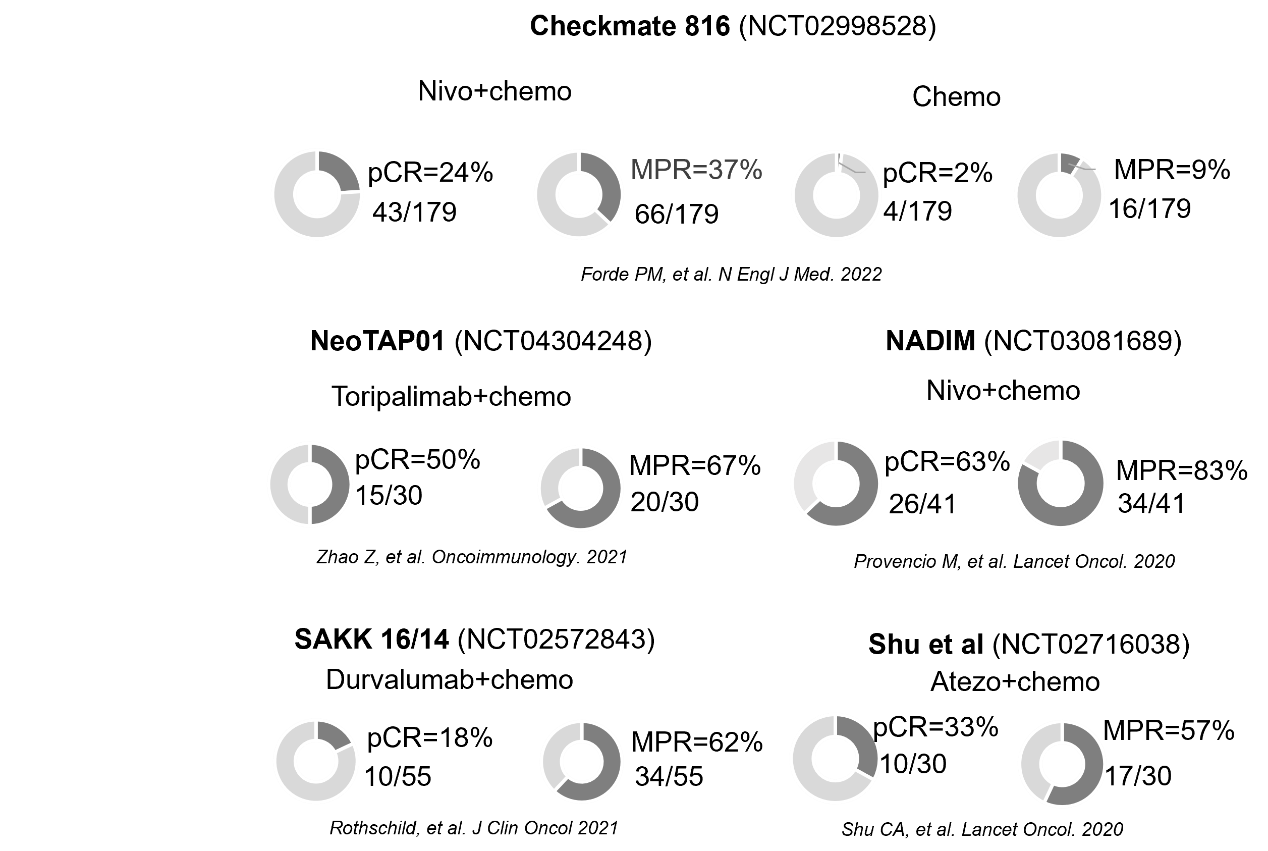


**Supplementary Figure 2 The pCR and MPR rates of NSCLC patients upon neoadjuvant therapy in clinical trials.** pCR, complete pathologic response; MPR, major pathologic response.


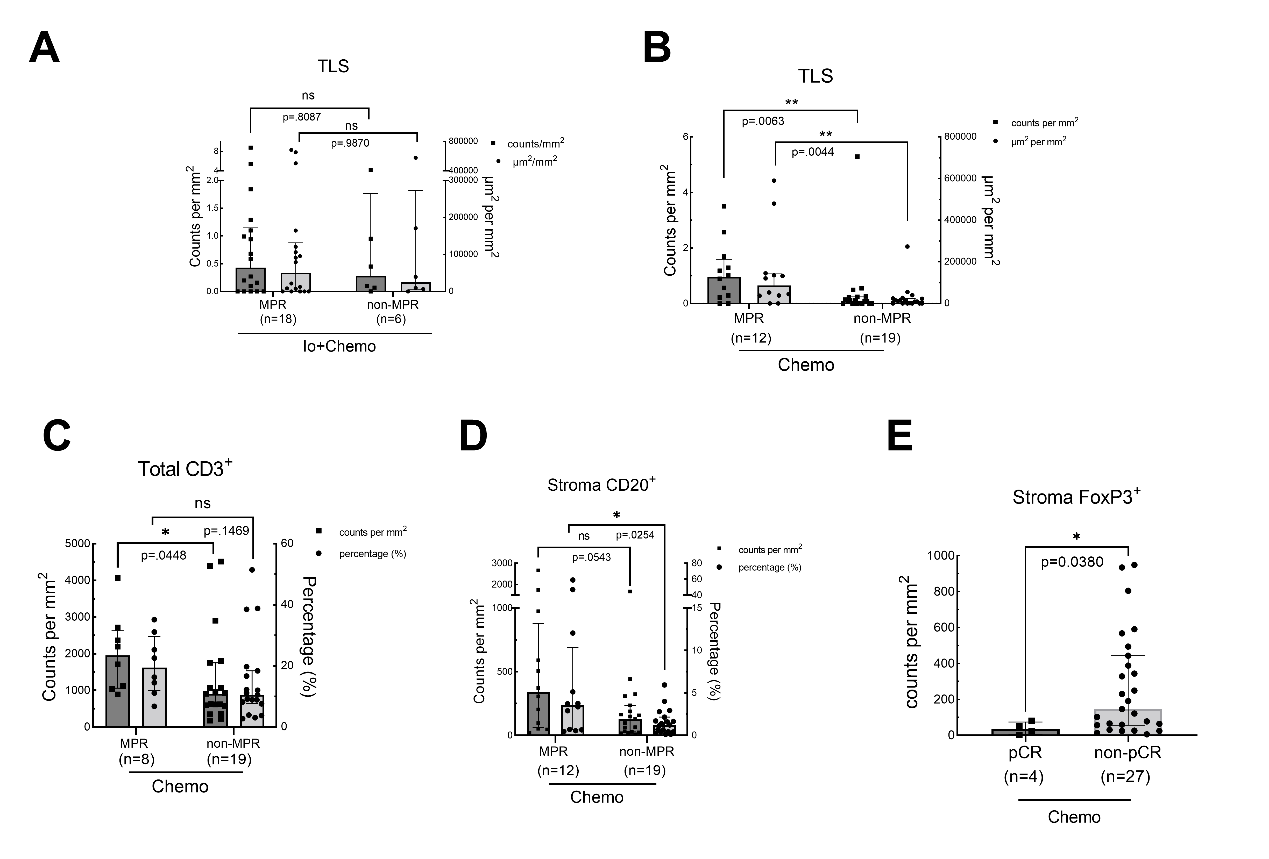


**Supplementary Figure 3 The association between immune cell infiltration in the TIME and pathologic response in treatment subgroups.** The scatter plot with bar was shown as median with interquartile range. Io+Chemo: immunochemotherapy; Chemo: chemotherapy; pCR: pathological complete response; MPR: major pathological response; **P* <0.05; ***P* <0.01; ns: no statistical significance.

Table S1 The immune cell subsets in patients with neoadjuvant immunochemotherapy compared with chemotherapy alone.

|  | NAT | (n=55) |  |  |  |  |  |  |  |  |  |  |  |  |
| --- | --- | --- | --- | --- | --- | --- | --- | --- | --- | --- | --- | --- | --- | --- |
|  | Io+Chemo | counts/mm^2^ |  | Chemo | (counts/mm^2^) |  | Io+chemo  vs chemo | Io+Chemo | (%) |  | Chemo | (%) |  | Io+chemo  vs chemo |
|  | Median | Min~Max | 25%; 75%  percentile | Median | Min~Max | 25%; 75%  percentile | P-value | Median | Min~Max | 25%; 75%  percentile | Median | Min~Max | 25%; 75%  percentile | P-value |
| CD3^+^  Tumor (n=17 vs 27)  Stroma (n=24 vs 31)  Total (n=17 vs 27) | 1285  1319  1204 | 229~3871  113~6879  165~6337 | 629; 3165  562; 2814  745; 3102 | 820  1169  1040 | 83~4882  87~8976  177~4514 | 398; 1460  478; 2946  619; 2194 | 0.3062  0.9664  0.4481 | 15.54  14.79  16.16 | 3.30~53.45  2.80~50.42  3.09~47.32 | 7.68; 30.58  9.63; 30.41  11.42; 31.64 | 10.40  14.08  12.01 | 1.19~65.59  0.71~50.24  2.76~51.45 | 5.16; 17.44  6.26; 31.14  8.15; 22.58 | 0.2503  0.5057  0.1870 |
| CD3^+^CD4^+^  Tumor (n=17 vs 27)  Stroma (n=24 vs 31)  Total (n=17 vs 27) | 201  309  275 | 14~1920  8~4303  13~3874 | 36; 540  47; 697  65; 625 | 87  176  131 | 0~1123  2~2338  1~1275 | 30; 222  49; 512  51; 348 | 0.1808  0.3601  0.2787 | 2.15  3.29  2.99 | 0.26~15.22  0.13~31.54  0.24~28.78 | 0.73; 5.48  0.95; 9.00  1.17; 8.03 | 0.96  1.62  1.95 | 0.00~11.86  0.02~16.12  0.01~11.02 | 0.40; 2.56  0.62; 4.25  0.59; 3.56 | 0.1619  0.1973  0.1512 |
| CD8^+^  Tumor (n=17 vs 27)  Stroma (n=24 vs 31)  Total (n=17 vs 27) | 248  332  310 | 17~1695  41~1709  57~1503 | 102; 484  148; 739  139; 1008 | 177  216  182 | 11~776  2~2178  9~1926 | 52; 369  68; 425  105; 451 | 0.3237  0.1328  0.2404 | 3.83  3.79  3.61 | 0.34~27.66  1.05~14.96  0.97~14.93 | 1.26; 5.97  2.19; 5.84  1.84; 9.60 | 2.70  2.12  2.58 | 0.17~16.69  0.02~20.60  0.17~20.35 | 0.64; 4.51  1.17; 4.29  1.03; 4.64 | 0.2238  **0.0335**  0.1348 |
| FoxP3^+^  Tumor (n=17 vs 27)  Stroma (n=24 vs 31)  Total (n=17 vs 27) | 57  80  85 | 8~3251  2~1577  6~2454 | 34; 335  32; 210  41; 252 | 81  102  167 | 1~577  0~948  4~682 | 31; 209  30; 388  32; 273 | 0.4772  0.3557  0.7161 | 1.13  1.20  1.50 | 0.22~49.76  0.07~18.66  0.15~32.95 | 0.33; 4.07  0.27; 2.74  0.41; 3.41 | 0.94  1.21  1.84 | 0.01~6.09  0.01~10.00  0.05~7.32 | 0.39; 3.02  0.30; 3.44  0.39; 3.07 | 0.8162  0.4764  0.8910 |
| PD-1^+^CD8^+^  Tumor (n=17 vs 27)  Stroma (n=24 vs 31)  Total (n=17 vs 27) | 3  9  5 | 0~208  0~1101  0~640 | 2; 9  2; 30  2; 21 | 2  4  3 | 0~419  0~824  0~710 | 0; 27  0; 51  1; 29 | 0.6681  0.3585  0.5770 | 0.07  0.08  0.07 | 0.00~3.05  0.00~9.67  0.00~7.08 | 0.02; 0.16  0.04; 0.39  0.03; 0.30 | 0.02  0.05  0.04 | 0.00~5.62  0.00~7.79  0.00~7.50 | 0.00; 0.38  0.01; 0.47  0.01; 0.36 | 0.6776  0.4097  0.5943 |
| CD3^+^CD4^+^FoxP3^+^  Tumor (n=17 vs 27)  Stroma (n=24 vs 31)  Total (n=17 vs 27) | 31  11  23 | 2~123  0~407  2~254 | 7; 72  4; 49  8; 55 | 11  22  25 | 0~261  0~378  0~193 | 4; 40  5; 88  9; 72 | 0.2835  0.5076  0.7429 | 0.24  0.19  0.23 | 0.04~1.76  0.00~4.82  0.04~3.41 | 0.12; 0.90  0.04; 0.54  0.13; 0.57 | 0.23  0.20  0.27 | 0.00~2.75  0.00~5.35  0.00~2.12 | 0.06; 0.43  0.06; 1.32  0.13; 1.12 | 0.4128  0.6521  0.7793 |
| CD68^+^CD163^+^  Tumor (n=17 vs 27)  Stroma (n=24 vs 31)  Total (n=17 vs 27) | 15  30  19 | 0~316  1~1033  1~792 | 6; 62  5; 151  4; 119 | 11  51  31 | 0~803  0~1753  0~1181 | 5; 35  7; 108  6; 76 | 0.7882  0.9899  0.9762 | 0.20  0.34  0.28 | 0.00~2.58  0.02~12.27  0.02~5.76 | 0.06; 0.93  0.09; 1.49  0.09; 1.16 | 0.15  0.53  0.35 | 0.00~7.82  0.00~19.71  0.00~12.16 | 0.09; 0.45  0.08; 1.22  0.09; 0.89 | 0.5870  0.8896  0.9477 |
| CD68^+^CD163^-^  Tumor (n=17 vs 27)  Stroma (n=24 vs 31)  Total (n=17 vs 27) | 70  167  127 | 12~1350  22~3477  21~3259 | 28; 216  81; 654  45; 554 | 47  151  130 | 1~1638  0~2493  9~1979 | 21; 136  48; 452  42; 273 | 0.2578  0.6893  0.7250 | 0.95  1.93  1.31 | 0.20~9.03  0.33~24.92  0.31~23.73 | 0.39; 3.86  0.98; 6.34  0.75; 5.37 | 0.75  1.78  1.29 | 0.01~15.97  0.00~28.02  0.12~20.36 | 0.33; 1.48  0.57; 4.21  0.49; 2.79 | 0.2579  0.4406  0.5468 |
| PD-L1^+^CD68^+^  Tumor (n=17 vs 27)  Stroma (n=24 vs 31)  Total (n=17 vs 27) | 2  1  1 | 0~21  0~181  0~94 | 0; 4  0; 7  1; 5 | 5  9  7 | 0~370  0~463  0~302 | 0; 13  3; 63  2; 37 | **0.0462**  **0.0147**  **0.0248** | 0.02  0.01  0.01 | 0.00~0.29  0.00~2.30  0.00~1.04 | 0.00; 0.06  0.00; 0.11  0.00; 0.07 | 0.07  0.09  0.08 | 0.00~3.66  0.00~4.03  0.00~3.08 | 0.00; 0.25  0.02; 0.79  0.03; 0.49 | 0.0537  **0.0171**  **0.0156** |
| CD56bright  Tumor (n=17 vs 27)  Stroma (n=24 vs 31)  Total (n=17 vs 27) | 1  28  2 | 0~347  0~595  0~486 | 0; 8  1; 124  0; 97 | 0  15  5 | 0~61  0~209  0~71 | 0; 7  0; 59  0; 19 | 0.5784  0.1896  0.8296 | 0.03  0.32  0.02 | 0.00~3.70  0.00~8.77  0.00~4.81 | 0.00; 0.10  0.01; 2.15  0.01; 1.23 | 0.01  0.17  0.05 | 0.00~0.86  0.00~3.86  0.00~1.03 | 0.00; 0.07  0.00; 0.53  0.00; 0.34 | 0.5096  0.1955  0.5477 |
| CD56dim  Tumor (n=17 vs 27)  Stroma (n=24 vs 31)  Total (n=17 vs 27) | 2  93  22 | 0~2424  0~2823  0~2127 | 0; 57  9; 944  2; 111 | 7  34  22 | 0~414  0~1093  0~415 | 0; 58  5; 314  3; 157 | 0.5723  0.5300  0.8344 | 0.06  1.03  0.52 | 0.00~25.87  0.00~30.23  0.00~21.07 | 0.00; 0.65  0.13; 11.61  0.02; 1.55 | 0.11  0.50  0.24 | 0.00~5.82  0.00~17.09  0.00~6.16 | 0.01; 0.79  0.04; 3.07  0.03; 2.11 | 0.6071  0.3466  0.9952 |
| CD20^+^  Tumor (n=17 vs 27)  Stroma (n=24 vs 31)  Total (n=17 vs 27) | 64  192  143 | 2~2738  4~2408  5~1278 | 5; 196  21; 508  22; 436 | 24  157  60 | 2~549  9~2664  3~949 | 10; 66  47; 372  32; 297 | 0.7701  0.9162  0.7611 | 0.61  2.56  1.39 | 0.04~27.72  0.07~12.94  0.11~9.49 | 0.07; 2.41  0.41; 5.24  0.29; 5.32 | 0.30  1.58  0.74 | 0.03~5.55  0.11~59.04  0.04~8.20 | 0.15; 1.40  0.55; 3.73  0.39; 2.82 | 0.7249  0.6524  0.5872 |

Table S2 TLS in patients with neoadjuvant immunochemotherapy compared with chemotherapy alone.

|  | NAT (n=55) |  |  |  |  |  |  |  |  |
| --- | --- | --- | --- | --- | --- | --- | --- | --- | --- |
|  | Io+Chemo | (n=24) |  |  | Chemo | (n=31) |  |  |  |
| TLS | Median | Min ~Max | 25% percentile | 75% percentile | Median | Min ~Max | 25% percentile | 75% percentile | P-value |
| counts/mm^2^ | 0.36 | 0.00~8.75 | 0.02 | 1.07 | 0.22 | 0.00~5.29 | 0.00 | 0.89 | 0.4726 |
| μm^2^/mm^2^ | 30468 | 0~684484 | 1594 | 153747 | 22634 | 0~589706 | 0.00 | 55207 | 0.5663 |

Abbreviation: TLS, tertiary lymphoid structures.

Table S3 The immune cell subsets in patients who reached pCR and non-pCR upon neoadjuvant therapy.

|  | NAT | (n=55) |  |  |  |  |  |  |  |  |  |  |  |  |
| --- | --- | --- | --- | --- | --- | --- | --- | --- | --- | --- | --- | --- | --- | --- |
|  | pCR | (counts/mm^2^) |  | non-pCR | (counts/mm^22^) |  | pCR vs  non-pCR | pCR | (%) |  | non-pCR | (%) |  | pCR vs  non-pCR |
|  | Median | Min~Max | 25%; 75%  percentile | Median | Min~Max | 25%; 75%  percentile | P-value | Median | Min~Max | 25%; 75%  percentile | Median | Min~Max | 25%; 75%  percentile | P-value |
| CD3^++^  Tumor (n=4 vs 40)  Stroma (n=12 vs 43)  Total (n=4 vs 40) | 1475  1963  3198 | 229~3941  113~5019  165~4090 | 493; 3372  241; 2838  802; 3988 | 812  1166  1044 | 83~4882  87~8976  177~6337 | 519; 2445  566; 2397  620; 2111 | 0.4918  0.8960  0.3290 | 13.77  23.37  28.44 | 3.30~28.21  2.80~37.53  3.09~36.47 | 5.90; 24.61  8.56; 31.44  8.75; 35.14 | 11.36  13.91  13.66 | 1.19~65.59  0.71~50.42  2.76~51.45 | 6.82; 29.62  7.15; 29.38  8.94; 25.07 | 0.9216  0.4997  0.4175 |
| CD3^+^CD4^++^  Tumor (n=4 vs 40)  Stroma (n=12 vs 43)  Total (n=4 vs 40) | 116  186  330 | 18~280  2~1381  13~948 | 37; 244  30; 925  23; 863 | 114  267  182 | 0~1920  3~4303  1~3874 | 30; 311  64; 543  59; 404 | 0.8654  0.9239  0.9482 | 1.08  2.16  2.22 | 0.26~2.33  0.10~15.07  0.24~9.40 | 0.37; 2.12  0.66; 8.37  0.34; 8.01 | 1.44  2.63  2.20 | 0.00~15.22  0.02~31.54  0.01~28.78 | 0.49; 3.75  0.86; 5.03  1.04; 4.40 | 0.5114  0.8367  0.8043 |
| CD8^++^  Tumor (n=4 vs 40)  Stroma (n=12 vs 43)  Total (n=4 vs 40) | 359  341  241 | 85~732  41~1709  57~1503 | 120; 672  69; 460  74; 1217 | 170  250  209 | 11~1695  2~2178  9~1926 | 69; 379  146; 475  114; 521 | 0.3268  0.8932  0.9843 | 2.89  3.29  2.51 | 0.98~5.51  1.17~10.82  1.10~9.84 | 1.36; 4.96  1.60; 4.96  1.18; 8.28 | 2.75  2.92  2.89 | 0.17~27.66  0.02~20.60  0.17~20.35 | 0.76; 5.36  1.26; 5.54  1.63; 5.47 | 0.9795  0.6327  0.9193 |
| FoxP3^++^  Tumor (n=4 vs 40)  Stroma (n=12 vs 43)  Total (n=4 vs 40) | 94  38  51 | 23~266  0~753  28~366 | 32; 232  24; 80  32; 289 | 69  121  140 | 1~3251  5~1577  4~2454 | 32; 243  41; 328  36; 258 | 0.8836  0.0539  0.5907 | 0.71  0.43  0.60 | 0.33~2.84  0.01~8.06  0.27~3.63 | 0.37; 2.37  0.24; 1.93  0.33; 2.89 | 1.13  1.53  1.63 | 0.01~49.76  0.04~18.66  0.05~32.95 | 0.38; 3.37  0.30; 3.21  0.43; 3.14 | 0.5609  0.1342  0.4389 |
| PD-1^+^CD8^+^  Tumor (n=4 vs 40)  Stroma (n=12 vs 43)  Total (n=4 vs 40) | 3  5  5 | 0 ~7  0~82  0~11 | 1; 6  2; 15  1; 10 | 3  6  5 | 0~419  0~1101  0~710 | 0; 24  1; 51  1; 29 | 0.6695  0.6679  0.5455 | 0.02  0.08  0.06 | 0.00~0.08  0.00~0.71  0.00~0.08 | 0.00; 0.07  0.04; 0.29  0.01; 0.08 | 0.06  0.07  0.07 | 0.00~5.62  0.00~9.67  0.00~7.50 | 0.00; 0.33  0.01; 0.47  0.01; 0.38 | 0.3074  0.9558  0.4932 |
| CD3^+^CD4^+^FoxP3^+^  Tumor (n=4 vs 40)  Stroma (n=12 vs 43)  Total (n=4 vs 40) | 14  7  9 | 3~42  0~73  2~57 | 4; 37  0; 17  3; 46 | 19  23  27 | 0~261  0~407  0~254 | 5; 55  9; 88  9; 68 | 0.6289  **0.0288**  0.2520 | 0.11  0.07  0.07 | 0.05~0.45  0.00~0.79  0.04~0.56 | 0.05; 0.38  0.01; 0.37  0.05; 0.44 | 0.24  0.24  0.27 | 0.00~2.75  0.00~5.35  0.00~3.41 | 0.10; 0.69  0.07; 1.32  0.14; 0.94 | 0.3473  0.0525  0.1016 |
| CD68^+^CD163^+^  Tumor (n=4 vs 40)  Stroma (n=12 vs 43)  Total (n=4 vs 40) | 6  20  13 | 0~316  1~178  1~159 | 1; 239  5; 131  2; 125 | 13  51  31 | 0~803  0~1753  0~1181 | 6; 39  7; 118  7; 78 | 0.4687  0.4110  0.4856 | 0.06  0.53  0.12 | 0.00~2.11  0.01~3.89  0.01~1.60 | 0.01; 1.60  0.09; 1.70  0.03; 1.24 | 0.20  0.52  0.34 | 0.00~7.82  0.00~19.71  0.00~12.16 | 0.09; 0.45  0.08; 1.22  0.11; 0.91 | 0.2868  0.9719  0.3675 |
| CD68^+^CD163^-^  Tumor (n=4 vs 40)  Stroma (n=12 vs 43)  Total (n=4 vs 40) | 49  173  99 | 14 ~1350  17~1503  37~1499 | 18; 1030  44; 355  38; 1163 | 55  164  129 | 1~1638  0~3477  9~3259 | 23; 143  88; 479  50; 282 | 0.9466  0.5972  0.9578 | 0.42  1.71  0.86 | 0.16~9.03  0.44~15.38  0.49~15.10 | 0.20; 6.90  0.64; 5.45  0.55; 11.58 | 0.88  1.78  1.40 | 0.01~15.97  0.00~28.02  0.12~23.73 | 0.35; 1.65  0.85; 5.04  0.76; 2.87 | 0.5389  0.8513  0.6248 |
| PD-L1^+^CD68^+^  Tumor (n=4 vs 40)  Stroma (n=12 vs 43)  Total (n=4 vs 40) | 0  1  1 | 0~3  0~52  0~1 | 0; 2  0; 5  0; 1 | 4  7  5 | 0~370  0~463  0~302 | 0; 10  1; 63  1; 37 | 0.0543  **0.0283**  **0.0473** | 0.00  0.01  0.01 | 0.00~0.02  0.00~0.49  0.00~0.01 | 0.00; 0.02  0.00; 0.18  0.00; 0.01 | 0.06  0.08  0.06 | 0.00~3.66  0.00~4.03  0.00~3.08 | 0.00; 0.18  0.01; 0.79  0.01; 0.43 | **0.0352**  0.0759  **0.0180** |
| CD56bright  Tumor (n=4 vs 40)  Stroma (n=12 vs 43)  Total (n=4 vs 40) | 0  58  7 | 0~347  0~545  0~486 | 0; 260  6; 398  0; 368 | 1  7  5 | 0~207  0~595  0~219 | 0; 7  0; 66  0; 43 | 0.6518  0.0591  0.9126 | 0.01  0.99  0.09 | 0.00~3.70  0.00~6.25  0.00~4.81 | 0.00; 2.78  0.11; 4.61  0.00; 3.65 | 0.01  0.11  0.05 | 0.00~1.57  0.00~8.77  0.00~2.27 | 0.00; 0.09  0.00; 0.78  0.00; 0.45 | 0.9238  **0.0135**  0.7286 |
| CD56dim  Tumor (n=4 vs 40)  Stroma (n=12 vs 43)  Total (n=4 vs 40) | 1  397  6 | 0~2424  1~2823  1~2127 | 0; 1818  15; 1251  1; 1598 | 7  34  24 | 0~414  0~1855  0~1280 | 0; 58  5; 237  3; 134 | 0.5708  **0.0398**  0.6477 | 0.01  10.23  0.08 | 0.00~25.87  0.01~30.23  0.00~21.07 | 0.00; 19.41  0.38; 16.71  0.01; 15.84 | 0.10  0.50  0.40 | 0.00~5.82  0.00~27.34  0.00~13.22 | 0.00; 0.65  0.04; 2.45  0.04; 1.64 | 0.5967  **0.0136**  0.6015 |
| CD20^+^  Tumor (n=4 vs 40)  Stroma (n=12 vs 43)  Total (n=4 vs 40) | 132  301  272 | 4~483  11~2408  8~627 | 21; 410  65; 894  61; 551 | 22  148  66 | 2~2738  4~2664  3~1278 | 9; 104  34; 372  25; 316 | 0.3684  0.1793  0.3991 | 1.33  3.46  2.55 | 0.06~3.46  0.27~59.04  0.15~6.22 | 0.20; 3.11  0.93; 10.13  0.46; 5.59 | 0.31  1.40  0.79 | 0.03~27.72  0.07~46.97  0.04~9.49 | 0.14; 1.45  0.43; 3.90  0.33; 3.19 | 0.4836  **0.0488**  0.5636 |

Table S4 The immune cell subsets in patients who reached MPR and non-MPR upon neoadjuvant therapy.

|  | NAT | (n=55) |  |  |  |  |  |  |  |  |  |  |  |  |
| --- | --- | --- | --- | --- | --- | --- | --- | --- | --- | --- | --- | --- | --- | --- |
|  | MPR | (counts/mm^2^) |  | non-MPR | (counts/mm^2^) |  | MPR vs  non-MPR | MPR | (%) |  | non-MPR | (%) |  | MPR vs  non-MPR |
|  | Median | Min~Max | 25%; 75%  percentile | Median | Min~Max | 25%; 75%  percentile | P-value | Median | Min~Max | 25%; 75%  percentile | Median | Min~Max | 25%; 75%  percentile | P-value |
| CD3^+^  Tumor (n=19 vs 25)  Stroma (n=30 vs 25)  Total (n=19 vs 25) | 1285  1519  1860 | 83~4536  113~7285  165~6337 | 640; 3661  639; 2907  1040; 3682 | 668  1045  917 | 118~4882  87~8976  177~4514 | 240; 1357  477; 2335  494; 1413 | **0.0491**  0.2856  **0.0090** | 13.82  20.11  22.58 | 1.19~53.45  2.80~50.42  3.09~47.32 | 7.87; 31.62  7.98; 32.01  13.49; 34.58 | 10.89  12.36  11.15 | 2.23~65.59  0.71~47.49  2.76~51.45 | 4.88; 16.89  6.63; 24.41  7.90; 19.11 | 0.2685  0.1145  **0.0218** |
| CD3^+^CD4^+^  Tumor (n=19 vs 25)  Stroma (n=30 vs 25)  Total (n=19 vs 25) | 212  309  332 | 16~1920  2~4303  13~3874 | 111; 572  47; 732  113; 641 | 48  145  131 | 0~698  3~1015  1~624 | 22; 201  44; 422  36; 246 | **0.0201**  0.1991  **0.0143** | 2.15  3.51  3.82 | 0.22~15.22  0.10~31.54  0.24~28.78 | 0.96; 6.74  1.02; 9.96  1.32; 9.40 | 0.88  1.69  1.95 | 0.00~7.94  0.02~9.60  0.01~7.11 | 0.44; 2.51  0.63; 3.28  0.57; 3.31 | 0.0764  0.1606  **0.0305** |
| CD8^+^  Tumor (n=19 vs 25)  Stroma (n=30 vs 25)  Total (n=19 vs 25) | 367  323  310 | 11~1695  41~1709  23~1503 | 97; 647  151; 486  138; 900 | 149  189  178 | 12~720  2~2178  9~1926 | 60; 308  84; 411  98; 1926 | 0.1767  0.1236  0.1430 | 2.99  3.79  3.61 | 0.17~27.66  0.52~14.96  0.28~14.93 | 0.99; 6.90  1.96; 5.64  1.41; 8.95 | 2.71  2.12  2.54 | 0.21~16.69  0.02~20.60  0.17~20.35 | 0.66; 4.63  1.04; 3.98  1.31; 4.17 | 0.5223  0.0585  0.2365 |
| FoxP3^+^  Tumor (n=19 vs 25)  Stroma (n=30 vs 25)  Total (n=19 vs 25) | 123  79  211 | 10~3251  1~1577  19~2454 | 37; 320  33; 283  42; 295 | 35  102  115 | 1~361  5~948  4~608 | 22; 199  27; 289  31; 199 | 0.0686  0.8177  0.1885 | 1.23  1.18  1.84 | 0.07~49.76  0.01~18.66  0.19~32.95 | 0.38; 4.06  0.31; 3.18  0.52; 3.52 | 0.93  1.32  1.53 | 0.01~5.06  0.04~8.97  0.05~6.68 | 0.37; 3.01  0.28; 3.18  0.36; 2.78 | 0.3812  0.9499  0.3877 |
| PD-1^+^CD8^+^  Tumor (n=19 vs 25)  Stroma (n=30 vs 25)  Total (n=19 vs 25) | 3  5  5 | 0~419  0~151  0~283 | 0; 27  2; 23  1; 24 | 3  6  5 | 0~208  0~1101  0~710 | 1; 12  1; 56  1; 31 | 0.9385  0.8433  0.8736 | 0.02  0.08  0.06 | 0.00~5.62  0.00~1.13  0.00~2.71 | 0.00; 0.38  0.02; 0.35  0.01; 0.21 | 0.05  0.08  0.09 | 0.00~3.18  0.00~9.67  0.00~7.50 | 0.01; 0.17  0.01; 0.56  0.01; 0.45 | 0.7445  0.8237  0.5922 |
| CD3^+^CD4^+^FoxP3^+^  Tumor (n=19 vs 25)  Stroma (n=30 vs 25)  Total (n=19 vs 25) | 31  14  34 | 0~261  0~407  0~254 | 11; 66  5; 77  10; 108 | 10  22  18 | 0~124  0~378  0~193 | 3; 46  4; 50  7; 58 | 0.0683  0.9031  0.2507 | 0.31  0.20  0.26 | 0.00~2.75  0.00~5.35  0.00~3.41 | 0.13; 1.02  0.04; 0.92  0.15; 1.36 | 0.21  0.19  0.20 | 0.00~2.02  0.00~3.58  0.00~2.12 | 0.06; 0.54  0.06; 0.64  0.13; 0.69 | 0.2362  0.9231  0.4073 |
| CD68^+^CD163^+^  Tumor (n=19 vs 25)  Stroma (n=30 vs 25)  Total (n=19 vs 25) | 16  64  52 | 0~316  0~964  0~792 | 5; 66  4; 153  4; 145 | 9  38  24 | 0~803  1~1753  1~1181 | 5; 38  12; 83  10; 39 | 0.7736  0.7917  0.6261 | 0.20  0.77  0.49 | 0.00~2.11  0.00~12.27  0.00~5.76 | 0.04; 0.89  0.08; 1.62  0.05; 1.41 | 0.18  0.34  0.28 | 0.00~7.82  0.02~19.71  0.01~12.16 | 0.09; 0.45  0.17; 0.85  0.12; 0.53 | 0.8834  0.6236  0.7739 |
| CD68^+^CD163^-^  Tumor (n=19 vs 25)  Stroma (n=30 vs 25)  Total (n=19 vs 25) | 108  173  155 | 7~1350  0~3477  22~3259 | 28; 293  92; 609  80; 483 | 44  131  111 | 1~1638  22~2493  9~1979 | 23; 76  51; 440  39; 159 | 0.0536  0.3576  0.0764 | 1.46  2.20  1.71 | 0.10~9.03  0.00~24.92  0.27~23.73 | 0.33; 4.07  0.85; 6.54  0.75; 5.60 | 0.66  1.69  1.26 | 0.01~15.97  0.29~28.02  0.12~20.36 | 0.35; 1.11  0.57; 3.38  0.50; 2.10 | 0.1549  0.2875  0.1446 |
| PD-L1^+^CD68^+^  Tumor (n=19 vs 25)  Stroma (n=30 vs 25)  Total (n=19 vs 25) | 2  3  4 | 0~370  0~271  0~302 | 0; 4  0; 9  1; 7 | 6  9  6 | 0~79  0~463  0~174 | 1; 13  1; 91  1; 47 | 0.1488  0.0550  0.2998 | 0.02  0.04  0.03 | 0.00~3.66  0.00~2.80  0.00~3.08 | 0.00; 0.07  0.00; 0.20  0.01; 0.12 | 0.06  0.08  0.07 | 0.00~0.77  0.00~4.03  0.00~2.18 | 0.01; 0.22  0.01; 1.09  0.02; 0.50 | 0.1391  0.0857  0.2111 |
| CD56bright  Tumor (n=19 vs 25)  Stroma (n=30 vs 25)  Total (n=19 vs 25) | 0  15  2 | 0~347  0~595  0~486 | 0; 8  1; 95  0; 46 | 1  15  5 | 0~73  0~209  0~113 | 0; 6  0; 65  0; 41 | 0.8556  0.4734  0.7762 | 0.00  0.19  0.02 | 0.00~3.70  0.00~8.77  0.00~4.81 | 0.00; 0.08  0.01; 1.90  0.00; 0.47 | 0.01  0.17  0.10 | 0.00~0.86  0.00~2.64  0.00~1.56 | 0.00; 0.10  0.00; 0.71  0.00; 0.52 | 0.7158  0.3109  0.8160 |
| CD56dim  Tumor (n=19 vs 25)  Stroma (n=30 vs 25)  Total (n=19 vs 25) | 6  97  12 | 0~2424  0~2823  0~2127 | 0; 111  6; 722  2; 139 | 5  39  25 | 0~414  0~944  0~415 | 0; 56  3; 256  2; 131 | 0.9857  0.2988  0.9953 | 0.08  1.22  0.13 | 0.00~25.87  0.00~30.23  0.00~21.07 | 0.00; 1.60  0.07; 13.22  0.02; 1.66 | 0.11  0.54  0.52 | 0.00~5.82  0.00~8.99  0.00~6.16 | 0.01; 0.65  0.02; 2.47  0.02; 1.84 | 0.8074  0.2141  0.8098 |
| CD20^+^  Tumor (n=19 vs 25)  Stroma (n=30 vs 25)  Total (n=19 vs 25) | 73  301  322 | 4~2738  4~2664  8~1278 | 16; 321  51; 605  39; 451 | 15  96  48 | 2~549  5~1670  3~500 | 7; 53  26; 213  18; 155 | **0.0080**  **0.0214**  **0.0120** | 0.71  3.55  3.19 | 0.06~27.72  0.07~59.04  0.15~9.49 | 0.19; 3.05  0.69; 5.68  0.37; 4.63 | 0.22  1.16  0.61 | 0.03~4.66  0.11~7.53  0.04~6.92 | 0.11; 0.83  0.39; 2.39  0.22; 2.06 | **0.0425**  **0.0062**  **0.0176** |

Table S5 TLS in responders compared with non-responders in patients with NAT.

|  | NAT | (n=55) |  |  |  |  |  |  |  |  |  |  |  |  |
| --- | --- | --- | --- | --- | --- | --- | --- | --- | --- | --- | --- | --- | --- | --- |
|  | pCR | (n=12) |  | Non-pCR | (n=43) |  |  | MPR | (n=30) |  | Non-MPR (n=25) | |  |  |
| TLS | Median | Min~Max | 25%; 75% percentile | Median | Min~Max | 25%; 75% percentile | P-value | Median | Min ~Max | 25%; 75% percentile | Median | Min~ Max | 25%; 75% percentile | P-value |
| counts/mm^2^ | 0.78 | 0.00~8.75 | 0.04; 1.29 | 0.20 | 0.00~5.45 | 0.00; 0.89 | 0.2188 | 0.64 | 0.00~8.75 | 0.08; 1.29 | 0.10 | 0.00~5.29 | 0.00; 0.35 | **0.0181** |
| μm^2^/mm^2^ | 62091 | 0~653220 | 2120; 129539 | 16244 | 0~684484 | 0; 106222 | 0.5256 | 65935 | 0~684484 | 5503; 137878 | 12598 | 0~578569 | 0; 33016 | **0.0433** |

Abbreviations: TLS, tertiary lymphoid structures

Table S6 The immune cell subsets in patients who reached pCR in comparison to non-pCR upon neoadjuvant immunochemotherapy.

|  | Io+Chemo | (n=24) |  |  |  |  |  |  |  |  |  |  |  |  |
| --- | --- | --- | --- | --- | --- | --- | --- | --- | --- | --- | --- | --- | --- | --- |
|  | pCR | (counts/mm^2^) |  | non-pCR | (counts/mm^2^) |  |  | pCR | (%) |  | Non-pCR | (%) |  |  |
|  | median | Min~Max | 25%; 75%  percentile | median | Min~Max | 25%; 75%  percentile | P-value | median | Min~Max | 25%; 75%  percentile | median | Min~Max | 25%; 75%  percentile | P-value |
| CD3^+^  Tumor (n=3 vs 14)  Stroma (n=8 vs 16)  Total (n=3 vs 14) | 1285  2811  3682 | 229~1664  113~5019  165~4090 | 229; 1664  440; 3563  165; 4090 | 1083  1014  1126 | 232~3871  207~6879  365~6337 | 648; 3490  562; 2174  831; 2483 | 0.5088  0.3826  0.7676 | 13.71  21.99  25.74 | 3.30~13.82  2.80~37.53  3.09~36.47 | 3.30; 13.82  8.56; 32.18  3.09; 36.47 | 17.87  14.20  15.07 | 4.00~53.45  3.49~50.42  6.74~47.32 | 7.83; 30.93  9.63; 28.20  12.06; 30.17 | 0.1971  0.7417  >0.9999 |
| CD3^+^CD4^+^  Tumor (n=3 vs 14)  Stroma (n=8 vs 16)  Total (n=3 vs 14) | 137  548  606 | 18~280  8~1381  13~948 | 18; 280  58; 992  13; 948 | 207  251  229 | 14~1920  8~4303  27~3874 | 40; 747  47; 545  71; 618 | 0.6044  0.4250  0.8426 | 1.47  4.06  3.82 | 0.26~2.33  0.20~15.07  0.24~9.40 | 0.26; 2.33  0.92; 10.99  0.24; 9.40 | 2.23  2.65  2.76 | 0.42~15.22  0.13~31.53  0.55~28.78 | 0.82; 7.04  1.00; 6.25  1.27; 7.45 | 0.3618  0.7875  0.8588 |
| CD8^+^  Tumor (n=3 vs 14)  Stroma (n=8 vs 16)  Total (n=3 vs 14) | 492  441  358 | 85~732  41~1709  57~1503 | 85; 732  144; 484  57; 1503 | 205  235  254 | 17~1695  109~1541  66~1182 | 105; 406  148; 862  139; 971 | 0.4956  0.6861  0.8588 | 3.29  4.01  3.61 | 0.98~5.51  1.26~10.82  1.10~9.84 | 0.98; 5.51  2.61; 5.20  1.10; 9.84 | 4.39  3.48  3.83 | 0.34~27.66  1.05~14.96  0.97~14.93 | 1.40; 7.42  2.09; 7.99  1.85; 9.60 | 0.6765  0.9404  0.9529 |
| FoxP3^+^  Tumor (n=3 vs 14)  Stroma (n=8 vs 16)  Total (n=3 vs 14) | 57  38  42 | 23~266  2~753  28~366 | 23; 266  32; 298  28; 366 | 89  103  100 | 8~3251  5~1577  6~2454 | 35; 352  25; 210  50; 246 | 0.7515  0.4896  0.7676 | 0.47  0.43  0.52 | 0.33~2.84  0.07~8.06  0.27~3.63 | 0.33; 2.84  0.25; 2.95  0.27; 3.63 | 1.18  1.59  1.55 | 0.22~49.76  0.07~18.66  0.15~32.95 | 0.32; 4.29  0.27; 2.74  0.74; 3.37 | 0.6912  0.5380  0.6632 |
| PD-1^+^CD8^+^  Tumor (n=3 vs 14)  Stroma (n=8 vs 16)  Total (n=3 vs 14) | 3  10  5 | 2~7  2~82  4~11 | 2; 7  4; 28  4; 11 | 3  12  8 | 0~208  0~1101  0~640 | 1; 19  1; 35  1; 27 | >0.9999  >0.9999  0.9324 | 0.02  0.08  0.07 | 0.01~0.08  0.03~0.55  0.05~0.08 | 0.01; 0.08  0.06; 0.29  0.05; 0.08 | 0.07  0.09  0.07 | 0.00~3.05  0.00~9.67  0.00~7.08 | 0.02; 0.33  0.02; 0.58  0.02; 0.43 | 0.4971  0.9167  0.9794 |
| CD3^+^CD4^+^FoxP3^+^  Tumor (n=3 vs 14)  Stroma (n=8 vs 16)  Total (n=3 vs 14) | 21  8  11 | 3~42  0~73  2~57 | 3; 42  0; 47  2; 57 | 32  17  27 | 2~123  0~407  2~254 | 8; 79  9; 49  9; 56 | 0.6162  0.1861  0.6191 | 0.17  0.05  0.07 | 0.05~0.45  0.00~0.79  0.04~0.56 | 0.05; 0.45  0.01; 0.48  0.04; 0.56 | 0.28  0.23  0.25 | 0.04~1.76  0.00~4.82  0.05~3.41 | 0.14; 0.94  0.12; 0.70  0.15; 0.64 | 0.4147  0.1161  0.2044 |
| CD68^+^CD163^+^  Tumor (n=3 vs 14)  Stroma (n=8 vs 16)  Total (n=3 vs 14) | 7  50  21 | 5~316  3~154  4~159 | 5; 316  5; 131  4; 159 | 16  28  19 | 0~201  1~1033  1~792 | 6; 51  6; 226  4; 110 | >0.9999  0.7991  0.8853 | 0.08  0.53  0.14 | 0.04~2.11  0.04~2.60  0.09~1.60 | 0.04; 0.08  0.09; 1.43  0.09; 1.60 | 0.23  0.34  0.30 | 0.00~2.58  0.02~12.27  0.02~5.76 | 0.10; 0.75  0.09; 1.49  0.07; 1.04 | 0.8412  0.9166  0.9397 |
| CD68^+^CD163^--^  Tumor (n=3 vs 14)  Stroma (n=8 vs 16)  Total (n=3 vs 14) | 69  196  155 | 28~1350  42~1503  37~1499 | 28; 1350  89; 355  37; 1499 | 71  147  122 | 12~707  22~3477  21~3259 | 26; 197  81; 860  49; 478 | 0.7676  0.5686  0.6897 | 0.52  2.20  1.01 | 0.32~9.03  0.60~15.38  0.71~15.10 | 0.32; 9.03  1.16; 5.45  0.71; 15.10 | 1.00  1.66  1.51 | 0.20~7.27  0.33~24.92  0.31~23.73 | 0.41; 3.72  0.88; 7.17  0.75; 5.00 | 0.9529  0.8340  >0.9999 |
| PD-L1^+^CD68^+^  Tumor (n=3 vs 14)  Stroma (n=8 vs 16)  Total (n=3 vs 14) | 0  0  1 | 0~3  0~52  0~1 | 0; 3  0; 5  0; 1 | 2  3  3 | 0~21  0~181  0~94 | 0; 5  0; 8  1; 6 | 0.3985  0.1201  0.3029 | 0.00  0.00  0.00 | 0.00~0.02  0.00~0.35  0.00~0.01 | 0.00; 0.02  0.00; 0.17  0.00; 0.01 | 0.02  0.03  0.03 | 0.00~0.29  0.00~2.30  0.00~1.04 | 0.00; 0.07  0.00; 0.11  0.01; 0.08 | 0.2485  0.1653  0.1206 |
| CD56bright  Tumor (n=3 vs 14)  Stroma (n=8 vs 16)  Total (n=3 vs 14) | 0  83  0 | 0~347  0~545  0~486 | 0; 347  11; 522  0; 486 | 2  6  4 | 0~207  0~595  0~219 | 0; 7  0; 98  0; 90 | 0.9412  0.2808  0.8735 | 0.01  1.44  0.01 | 0.00~3.70  0.00~6.26  0.00~4.81 | 0.00; 3.70  0.12; 5.48  0.00; 4.81 | 0.03  0.12  0.05 | 0.00~1.57  0.00~8.77  0.00~2.27 | 0.00; 0.10  0.00; 1.53  0.01; 1.07 | 0.8412  0.1899  >0.9999 |
| CD56dim  Tumor (n=3 vs 14)  Stroma (n=8 vs 16)  Total (n=3 vs 14) | 1  692  1 | 0~2424  1~2823  1~2127 | 0; 2424  44; 1902  1; 2127 | 4  44  41 | 0~300  0~1855  0~1280 | 0; 57  9; 153  6; 109 | 0.8838  0.1166  0.7397 | 0.02  10.24  0.02 | 0.00~25.87  0.01~30.23  0.00~21.07 | 0.00; 25.87  0.29; 19.29  0.00; 21.07 | 0.08  0.60  0.59 | 0.00~3.04  0.00~27.34  0.00~13.22 | 0.00; 0.64  0.13; 2.21  0.09; 1.53 | 0.8838  0.1396  0.6941 |
| CD20^+^  Tumor (n=3 vs 14)  Stroma (n=8 vs 16)  Total (n=3 vs 14) | 73  397  221 | 4~191  11~2408  8~627 | 4; 191  81; 905  8; 627 | 40  123  108 | 2~2738  4~1481  5~1278 | 6; 228  20; 414  23; 404 | 0.8809  0.1493  0.8441 | 0.61  4.30  1.39 | 0.06~2.04  0.27~12.94  0.15~6.22 | 0.06; 2.04  0.93; 10.13  0.15; 6.22 | 0.51  1.95  1.58 | 0.04~27.72  0.07~10.86  0.11~9.49 | 0.08; 2.85  0.27; 4.57  0.30; 4.92 | 0.8574  0.1325  0.9397 |

Table S7 The immune cell subsets in patients who reached MPR in comparison to non-MPR upon neoadjuvant immunochemotherapy.

|  | Io+Chemo | (n=24) |  |  |  |  |  |  |  |  |  |  |  |  |
| --- | --- | --- | --- | --- | --- | --- | --- | --- | --- | --- | --- | --- | --- | --- |
|  | MPR | counts/mm^2^ |  | non-MPR | counts/mm^2^ |  |  | MPR | (%) |  | Non-MPR | (%) |  |  |
|  | median | Min~Max | 25%; 75%  percentile | median | Min~Max | 25%; 75%  percentile | P-value | median | Min~Max | 25%; 75%  percentile | median | Min~Max | 25%; 75%  percentile | P-value |
| CD3^+^  Tumor (n=11 vs 6)  Stroma (n=18 vs 6)  Total (n=11 vs 6) | 1664  1724  1860 | 229~3871  113 ~6879  165~6337 | 697; 3584  535; 3084  952; 4090 | 736  874  965 | 232~2681  476~2272  365~2522 | 243; 1692  540; 1352  400; 1416 | 0.1802  0.1994  0.1215 | 13.82  18.66  25.74 | 3.30~53.45  2.80~50.42  3.09~47.32 | 7.98; 31.62  9.55; 32.82  13.49; 36.47 | 15.94  12.26  13.26 | 4.00~30.46  8.93~24.64  6.74~27.44 | 6.54; 22.17  9.25; 23.53  8.00; 25.06 | 0.6605  0.3104  0.1490 |
| CD3^+^CD4^+^  Tumor (n=11 vs 6)  Stroma (n=18 vs 6)  Total (n=11 vs 6) | 245  396  412 | 18~1920  8~4303  13~3874 | 116; 892  64; 798  113; 760 | 36  121  118 | 14~698  37~474  27~611 | 17; 325  38; 269  33; 289 | 0.0642  0.1949  0.0932 | 2.33  4.31  4.26 | 0.26~15.22  0.13~31.54  0.24~28.78 | 1.46; 6.74  1.11; 10.71  1.65; 9.85 | 0.73  1.98  1.69 | 0.42~7.94  0.61~4.83  0.55~6.65 | 0.44; 3.71  0.80; 3.21  0.77; 3.55 | 0.1490  0.2796  0.0983 |
| CD8^+^  Tumor (n=11 vs 6)  Stroma (n=18 vs 6)  Total (n=11 vs 6) | 367  389  358 | 38~1695  41~1709  57~1503 | 97; 647  196; 835  154; 1105 | 191  168  169 | 17 ~476  109~1541  66~935 | 85; 394  125; 550  98; 479 | 0.4180  0.1593  0.2161 | 3.29  4.39  5.23 | 0.34~27.66  1.05~14.96  1.00~14.93 | 0.99; 10.62  2.43; 6.62  1.81; 9.84 | 4.39  2.71  2.90 | 0.41~6.35  1.26~13.54  0.97~10.35 | 1.36; 5.62  1.89; 5.64  1.64; 5.89 | 0.8836  0.3166  0.4623 |
| FoxP3^+^  Tumor (n=11 vs 6)  Stroma (n=18 vs 6)  Total (n=11 vs 6) | 129  73  114 | 23~3251  2~1577  19~2454 | 35; 350  33; 231  40; 283 | 46  104  82 | 8~357  6~194  6~263 | 13; 181  17; 142  41; 152 | 0.2250  0.9225  0.5249 | 1.23  0.97  1.50 | 0.28~49.76  0.07~18.66  0.27~32.95 | 0.33; 4.95  0.29; 3.18  0.30; 3.52 | 1.09  1.66  1.30 | 0.22~4.07  0.15~2.90  0.15~3.48 | 0.25; 2.05  0.19; 2.19  0.71; 2.11 | 0.3905  0.9634  0.6420 |
| PD-1^+^CD8^+^  Tumor (n=11 vs 6)  Stroma (n=18 vs 6)  Total (n=11 vs 6) | 3  9  5 | 0~73  1~84  1~83 | 2; 10  3; 23  1; 15 | 3  13  11 | 0~208  0~1101  0~640 | 1; 56  2; 305  2; 186 | 0.8230  0.8074  0.7512 | 0.07  0.08  0.07 | 0.00~1.52  0.00~1.12  0.00~1.13 | 0.01; 0.14  0.05; 0.24  0.02; 0.17 | 0.06  0.24  0.23 | 0.00~3.05  0.00~9.67  0.00~7.08 | 0.02; 0.90  0.03; 2.90  0.02; 2.19 | 0.8234  0.6136  0.5715 |
| CD3^+^CD4^+^FoxP3^+^  Tumor (n=11 vs 6)  Stroma (n=18 vs 6)  Total (n=11 vs 6) | 32  15  23 | 3~123  0~407  2~254 | 21; 66  2; 61  10; 57 | 7  10  19 | 2~80  2~52  2~63 | 2; 79  8; 38  5; 56 | 0.1877  0.9358  0.4779 | 0.31  0.20  0.23 | 0.05~1.76  0.00~4.82  0.04~3.41 | 0.17; 1.02  0.04; 0.63  0.15; 0.56 | 0.14  0.18  0.28 | 0.04~0.91  0.05~0.78  0.05~0.83 | 0.05; 0.90  0.09; 0.53  0.10; 0.64 | 0.2254  0.9099  0.7885 |
| CD68^+^CD163^+^  Tumor (n=11 vs 6)  Stroma (n=18 vs 6)  Total (n=11 vs 6) | 16  64  21 | 0~316  3~964  3~792 | 5; 84  8; 153  4; 159 | 11  20  19 | 1~129  1~1033  1~520 | 6; 62  3; 285  3; 157 | 0.8277  0.3686  0.4777 | 0.20  0.77  0.32 | 0.00~2.11  0.04~12.27  0.04~5.76 | 0.04; 1.27  0.10; 1.52  0.09; 1.41 | 0.23  0.30  0.27 | 0.02~2.58  0.02~9.07  0.02~5.76 | 0.14; 1.08  0.06; 2.52  0.07; 1.89 | 0.6781  0.4457  0.6799 |
| CD68^+^CD163^-^  Tumor (n=11 vs 6)  Stroma (n=18 vs 6)  Total (n=11 vs 6) | 70  184  155 | 19~1350  37~3477  37~3259 | 37; 348  127; 787  111; 707 | 49  83  66 | 12~178  22~1410  21~718 | 20; 105  23; 432  23; 267 | 0.2561  0.0559  0.0938 | 0.95  2.30  1.71 | 0.20~9.03  0.60~24.92  0.62~23.73 | 0.35; 6.33  1.23; 6.72  0.94; 6.12 | 0.86  0.95  0.93 | 0.29~3.57  0.33~12.38  0.31~7.95 | 0.40; 1.70  0.47; 4.36  0.45; 3.44 | 0.5908  0.0769  0.2561 |
| PD-L1^+^CD68^+^  Tumor (n=11 vs 6)  Stroma (n=18 vs 6)  Total (n=11 vs 6) | 2  1  1 | 0~21  0~181  0~50 | 0; 4  0; 6  1; 6 | 2  2  1 | 0~13  0~181  0~94 | 1; 5  0; 51  0; 27 | 0.9051  0.7985  0.6427 | 0.01  0.01  0.01 | 0.00~0.29  0.00~2.30  0.00~0.58 | 0.00; 0.07  0.00; 0.15  0.00; 0.06 | 0.02  0.02  0.02 | 0.00~0.19  0.00~1.59  0.00~1.04 | 0.01; 0.08  0.00; 0.46  0.00; 0.31 | 0.6302  0.9130  0.9837 |
| CD56bright  Tumor (n=11 vs 6)  Stroma (n=18 vs 6)  Total (n=11 vs 6) | 0  47  1 | 0~347  0~595  0~486 | 0; 8  1; 299  0; 111 | 2  6  5 | 0~73  0~128  0~113 | 0; 22  1; 106  0; 91 | >0.9999  0.6240  0.7783 | 0.01  0.54  0.01 | 0.00~3.70  0.00~8.77  0.00~4.81 | 0.00; 0.10  0.01; 3.03  0.00; 1.72 | 0.03  0.12  0.10 | 0.00~0.83  0.00~1.70  0.00~1.56 | 0.00; 0.28  0.01; 1.18  0.01; 1.07 | 0.9067  0.4920  0.7274 |
| CD56dim  Tumor (n=11 vs 6)  Stroma (n=18 vs 6)  Total (n=11 vs 6) | 1  144  14 | 0~2424  0~2823  0~2127 | 0; 281  7; 1290  1; 113 | 5  44  41 | 0~58  0~166  0~119 | 0; 57  8; 123  5; 101 | 0.9576  0.2861  0.9826 | 0.02  1.26  0.13 | 0.00~25.87  0.00~30.23  0.00~21.07 | 0.00; 2.13  0.11; 14.11  0.02; 1.66 | 0.13  0.60  0.59 | 0.00~0.65  0.00~2.48  0.00~1.57 | 0.00; 0.64  0.14; 1.69  0.11; 1.38 | 0.9576  0.4244  0.9409 |
| CD20^+^  Tumor (n=11 vs 6)  Stroma (n=18 vs 6)  Total (n=11 vs 6) | 191  286  221 | 4~2738  4~2408  8~1278 | 6; 321  37; 546  23; 593 | 14  36  24 | 2~99  5~568  5~500 | 3; 36  13; 273  8; 232 | 0.0576  0.1805  0.1722 | 1.47  3.53  3.19 | 0.06~27.72  0.07~12.94  0.15~9.49 | 0.06; 3.05  0.66; 5.68  0.31; 6.13 | 0.20  0.52  0.36 | 0.04~1.13  0.11~7.53  0.11~6.92 | 0.07; 0.52  0.20; 3.84  0.16; 3.14 | 0.1207  0.1949  0.2071 |

Table S8 TLS in responders compared with non-responders in patients with adjuvant immunochemotherapy.

|  | Io+Chemo | (n=24) |  |  |  |  |  |  |  |  |  |  |  |  |
| --- | --- | --- | --- | --- | --- | --- | --- | --- | --- | --- | --- | --- | --- | --- |
|  | pCR | (n=8) |  | Non-pCR | (n=16) |  |  | MPR | (n=18) |  | Non-MPR | (n=6) |  |  |
| TLS | Median | Min~Max | 25%; 75% percentile | Median | Min~Max | 25%; 75% percentile | P-value | Median | Min~Max | 25%; 75% percentile | Median | Min~Max | 25%; 75% percentile | P-value |
| counts/mm^2^ | 0.63 | 0.00~8.75 | 0.04; 1.24 | 0.33 | 0.00~5.45 | 0.02; 0.95 | 0.5551 | 0.43 | 0.00~8.75 | 0.00; 1.15 | 0.28 | 0.00~4.21 | 0.05; 1.77 | 0.8087 |
| μm^2^/mm^2^ | 50307 | 0~653220 | 2120; 146403 | 25984 | 0~684484 | 1594; 158346 | 0.9658 | 50307 | 0~684484 | 0; 131738 | 24503 | 0~578569 | 4782; 272805 | 0.9870 |

Abbreviation: TLS, tertiary lymphoid structures

Table S9 The immune cell subsets in patients who reached pCR in comparison to non-pCR upon neoadjuvant chemotherapy.

|  | Chemo | (n=31) |  |  |  |  |  |  |  |  |  |  |  |  |
| --- | --- | --- | --- | --- | --- | --- | --- | --- | --- | --- | --- | --- | --- | --- |
|  | pCR | counts/mm^2^ |  | non-pCR | (counts/mm^2^) |  |  | pCR | (%) |  | Non-pCR | (%) |  |  |
|  | median | Min~Max | 25%; 75%  percentile | median | Min~Max | 25%; 75%  percentile | P-value | median | Min~Max | 25%; 75%  percentile | median | Min~Max | 25%; 75%  percentile | P-value |
| CD3^+^  Tumor (n=1 vs 26)  Stroma (n=4 vs 27)  Total (n=1 vs 26) | 3941  890  2714 | 3941~3941  169~2582  2714~2714 | 3941; 3941  241; 2273  2714; 2714 | 730  1169  996 | 83~4882  87~8976  177~4514 | 356; 1379  663; 3091  613; 1899 | —  0.4078  — | 28.21  23.37  31.13 | 28.21~28.21  4.82~31.67  31.13~31.13 | 28.21; 28.21  8.40; 30.65  31.13; 31.13 | 10.29  13.91  11.58 | 1.19~65.59  0.71~50.24  2.76~51.45 | 5.02; 16.65  6.26; 31.14  8.02; 20.47 | —  0.6701  — |
| CD3^+^CD4^+^  Tumor (n=1 vs 26)  Stroma (n=4 vs 27)  Total (n=1 vs 26) | 94  44  54 | 94~94  2~219  54~54 | 94; 94  11; 177  54; 54 | 81  267  148 | 0~1123  3~2338  1~1275 | 27; 239  64; 543  49; 356 | —  0.0803  — | 0.68  0.85  0.62 | 0.68~0.68  0.10~3.14  0.62~0.62 | 0.68; 0.68  0.23; 2.63  0.62; 0.62 | 1.15  1.69  1.96 | 0.00~11.86  0.02~16.12  0.01~11.02 | 0.39; 2.77  0.63; 5.03  0.55; 3.67 | —  0.1927  — |
| CD8^+^  Tumor (n=1 vs 26)  Stroma (n=4 vs 27)  Total (n=1 vs 26) | 226  84  123 | 226~226  59~327  123~123 | 226; 226  61; 271  123; 123 | 163  264  201 | 11~776  2~2178  9~1926 | 49; 387  79; 428  101; 468 | —  0.2880  — | 2.49  2.12  1.41 | 2.49~2.49  1.17~4.29  1.41~1.41 | 2.49; 2.49  1.20; 3.96  1.41; 1.41 | 2.71  2.12  2.71 | 0.17~16.69  0.02~20.60  0.17~20.35 | 0.62; 4.57  0.88; 4.46  1.02; 4.72 | —  0.9321  — |
| FoxP3^+^  Tumor (n=1 vs 26)  Stroma (n=4 vs 27)  Total (n=1 vs 26) | 131  37  59 | 131~131  1~81  59~59 | 131; 131  6; 74  59; 59 | 64  146  171 | 1~577  5~948  4~682 | 30; 215  56; 443  32; 279 | —  **0.0380**  — | 0.94  0.47  0.68 | 0.94~0.94  0.01~2.31  0.68~0.68 | 0.94; 0.94  0.09; 1.89  0.68; 0.68 | 1.03  1.32  1.90 | 0.01~6.09  0.04~10.00  0.05~7.32 | 0.39; 3.07  0.30; 3.95  0.38; 3.09 | —  0.1748  — |
| PD-1^+^CD8^+^  Tumor (n=1 vs 26)  Stroma (n=4 vs 27)  Total (n=1 vs 26) | 0  3  0 | 0~0  0~16  0~0 | 0; 0  0; 13  0; 0 | 3  4  4 | 0~419  0~824  0~710 | 0; 30  1; 54  1; 34 | —  0.3633  — | 0.00  0.06  0.00 | 0.00~0.00  0.00~0.71  0.00~0.00 | 0.00; 0.00  0.00; 0.56  0.00; 0.00 | 0.04  0.05  0.07 | 0.00~5.62  0.00~7.79  0.00~7.50 | 0.00; 0.41  0.01; 0.47  0.01; 0.40 | —  0.5779  — |
| CD3^+^CD4^+^FoxP3^+^  Tumor (n=1 vs 26)  Stroma (n=4 vs 27)  Total (n=1 vs 26) | 7  6  6 | 7~7  0~15  6~6 | 7; 7  2; 13  6; 6 | 14  28  27 | 0~261  0~378  0~193 | 4; 43  5; 160  9; 81 | —  0.1033  — | 0.05  0.09  0.07 | 0.05~0.05  0.01~0.43  0.07~0.07 | 0.05; 0.05  0.03; 0.35  0.07; 0.07 | 0.24  0.31  0.28 | 0.00~2.75  0.00~5.35  0.00~2.12 | 0.08; 0.48  0.06; 1.53  0.14; 1.14 | —  0.3519  — |
| CD68^+^CD163^+^  Tumor (n=1 vs 26)  Stroma (n=4 vs 27)  Total (n=1 vs 26) | 0  24  1 | 0~0  1~178  1~1 | 0; 0  3; 143  1; 1 | 12  52  31 | 0~803  0~1753  0~1181 | 5; 38  7; 108  11; 87 | —  0.4833  — | 0.00  0.93  0.01 | 0.00~0.00  0.01~3.89  0.01~0.01 | 0.00; 0.00  0.04; 3.35  0.01; 0.01 | 0.16  0.53  0.36 | 0.00~7.82  0.00~19.71  0.00~12.16 | 0.09; 0.45  0.08; 1.21  0.14; 0.97 | —  0.9655  — |
| CD68^+^CD163^-^  Tumor (n=1 vs 26)  Stroma (n=4 vs 27)  Total (n=1 vs 26) | 14  41  42 | 14~14  17~496  42~42 | 14; 14  21; 384  42; 42 | 48  168  132 | 1~1638  0~2493  9~1979 | 22; 138  101; 452  47; 276 | —  0.1927  — | 0.16  0.67  0.49 | 0.16~0.16  0.44~10.81  0.49~0.49 | 0.16; 0.16  0.47; 8.30  0.49; 0.49 | 0.76  1.98  1.39 | 0.01~15.97  0.00~28.02  0.12~20.36 | 0.34; 1.52  0.80; 4.21  0.69; 2.82 | —  0.3793  — |
| PD-L1^+^CD68^+^  Tumor (n=1 vs 26)  Stroma (n=4 vs 27)  Total (n=1 vs 26) | 0  3  1 | 0~0  1~11  1~1 | 0; 0  2; 9  1; 1 | 6  9  9 | 0~370  0~463  0~302 | 1; 14  3; 78  2; 42 | —  0.2803  — | 0.00  0.05  0.01 | 0.00~0.00  0.01~0.49  0.01~0.01 | 0.00; 0.00  0.02; 0.38  0.01; 0.01 | 0.07  0.09  0.09 | 0.00~3.66  0.00~4.03  0.00~3.08 | 0.01; 0.27  0.02; 1.02  0.03; 0.50 | —  0.4837  — |
| CD56bright  Tumor (n=1 vs 26)  Stroma (n=4 vs 27)  Total (n=1 vs 26) | 0  40  14 | 0~0  3~95  14~14 | 0; 0  6; 87  14; 14 | 1  12  5 | 0~61  0~209  0~71 | 0; 8  0; 51  0; 26 | —  0.2861  — | 0.00  0.78  0.17 | 0.00~0.00  0.08~3.86  0.17~0.17 | 0.00; 0.00  0.11; 3.24  0.17; 0.17 | 0.01  0.11  0.05 | 0.00~0.86  0.00~2.64  0.00~1.03 | 0.00; 0.08  0.00; 0.46  0.00; 0.35 | —  0.0972  — |
| CD56dim  Tumor (n=1 vs 26)  Stroma (n=4 vs 27)  Total (n=1 vs 26) | 0  311  11 | 0~0  12~1093  11~11 | 0; 0  15; 969  11; 11 | 8  34  24 | 0~414  0~944  0~415 | 0; 62  3; 306  3; 164 | —  0.2098  — | 0.00  8.49  0.13 | 0.00~0.00  0.15~17.09  0.13~0.13 | 0.00; 0.00  0.46; 16.71  0.13; 0.13 | 0.11  0.40  0.26 | 0.00~5.82  0.00~8.99  0.00~6.16 | 0.01; 0.89  0.02; 2.45  0.03; 2.12 | —  0.1048  — |
| CD20^+^  Tumor (n=1 vs 26)  Stroma (n=4 vs 27)  Total (n=1 vs 26) | 483  255  323 | 483~483  19~975  323~323 | 483; 483  65; 808  323; 323 | 22  148  55 | 2~549  9~2664  3~949 | 10; 56  47; 372  32; 227 | —  0.6650  — | 3.46  3.33  3.71 | 3.46~3.46  0.55~59.04  3.71~3.71 | 3.46; 3.46  1.14; 45.22  3.71; 3.71 | 0.27  1.40  0.69 | 0.03~5.55  0.11~46.97  0.04~8.20 | 0.15; 0.99  0.47; 3.37  0.40; 2.80 | —  0.2373  — |

Table S10 The immune cell subsets in patients who reached MPR in comparison to non-MPR upon neoadjuvant chemotherapy.

|  | Chemo | (n=31) |  |  |  |  |  |  |  |  |  |  |  |  |
| --- | --- | --- | --- | --- | --- | --- | --- | --- | --- | --- | --- | --- | --- | --- |
|  | MPR | (counts/mm^2^) |  | non-MPR | (counts/mm^2^) |  |  | MPR | (%) |  | Non-MPR | (%) |  |  |
|  | median | Min~max | 25%; 75%  percentile | median | Min~max | 25%; 75%  percentile | P-value | median | Min~max | 25%; 75%  percentile | median | Min~max | 25%; 75%  percentile | P-value |
| CD3^+^  Tumor (n=8 vs 19)  Stroma (n=12 vs 19)  Total (n=8 vs 19) | 1032  1407  1953 | 83~4536  169~7285  888~4070 | 606; 3871  671; 2964  1061; 2627 | 614  1166  908 | 118~4882  87~8976  177~4514 | 229; 1352  340; 2946  577; 1754 | 0.3074  0.7342  **0.0448** | 13.28  20.11  19.46 | 1.19~38.67  4.82~50.24  6.78~35.16 | 7.41; 31.95  7.08; 31.77  11.96; 29.68 | 10.40  12.96  10.55 | 2.23~65.59  0.71~47.49  2.76~51.45 | 4.60; 15.67  4.48; 27.88  7.64; 18.45 | 0.6210  0.2198  0.1469 |
| CD3^+^CD4^+^  Tumor (n=8 vs 19)  Stroma (n=12 vs 19)  Total (n=8 vs 19) | 122  198  230 | 16~1123  2~2338  51~1275 | 37; 485  41; 512  71; 619 | 55  145  131 | 0~583  3~1015  1~624 | 30; 201  49; 512  37; 255 | 0.3389  0.9442  0.1758 | 1.16  1.47  2.24 | 0.22~11.86  0.10~16.12  0.44~11.02 | 0.34; 6.60  0.73; 7.15  0.78; 7.69 | 0.88  1.69  1.95 | 0.00~7.53  0.02~9.60  0.01~7.11 | 0.40; 2.55  0.62; 3.34  0.44; 3.34 | 0.5845  0.8262  0.3532 |
| CD8^+^  Tumor (n=8 vs 19)  Stroma (n=12 vs 19)  Total (n=8 vs 19) | 297  267  254 | 11~776  59~1371  23~900 | 43; 616  76; 438  110; 538 | 149  200  178 | 12~720  2~2178  9~1926 | 52; 249  58; 425  87; 429 | 0.5491  0.6458  0.5491 | 2.74  2.94  2.89 | 0.17~10.43  0.52~10.71  0.28~8.95 | 0.59; 6.50  1.21; 4.76  1.09; 5.40 | 2.70  1.90  2.34 | 0.21~16.69  0.02~20.60  0.17~20.35 | 0.64; 4.43  0.73; 4.25  1.03; 3.93 | 0.8872  0.3889  0.7262 |
| FoxP3^+^  Tumor (n=8 vs 19)  Stroma (n=12 vs 19)  Total (n=8 vs 19) | 114  101  226 | 10~577  1~934  22~682 | 52; 217  32; 468  69; 339 | 35  102  140 | 1~361  5~948  4~608 | 27; 209  30; 343  30; 206 | 0.3009  >0.9999  0.2023 | 1.20  1.71  2.20 | 0.07~6.09  0.01~10.00  0.19~7.32 | 0.64; 2.94  0.34; 3.27  0.77; 3.50 | 0.84  1.05  1.53 | 0.01~5.06  0.04~8.97  0.05~6.68 | 0.38; 3.02  0.28; 3.95  0.33; 3.06 | 0.5223  0.9284  0.4493 |
| PD-1^+^CD8^+^  Tumor (n=8 vs 19)  Stroma (n=12 vs 19)  Total (n=8 vs 19) | 1  3  2 | 0~419  0~151  0~283 | 0; 68  1; 27  0; 48 | 3  4  5 | 0~119  0~824  0~710 | 0; 16  0; 57  1; 28 | 0.7001  0.5786  0.8643 | 0.01  0.04  0.02 | 0.00~5.62  0.00~1.13  0.00~2.71 | 0.00; 0.70  0.00; 0.41  0.00; 0.50 | 0.05  0.05  0.09 | 0.00~3.18  0.00~7.79  0.00~7.50 | 0.01; 0.16  0.01; 0.48  0.01; 0.33 | 0.6249  0.6948  0.7423 |
| CD3^+^CD4^+^FoxP3^+^  Tumor (n=8 vs 19)  Stroma (n=12 vs 19)  Total (n=8 vs 19) | 20  14  71 | 0~261  0~303  0~139 | 5; 113  6; 156  12; 129 | 11  23  18 | 0~124  0~378  0~193 | 3; 40  3; 86  9; 66 | 0.5929  0.9442  0.3136 | 0.23  0.22  0.71 | 0.00~2.75  0.00~5.35  0.00~1.57 | 0.05; 1.56  0.07; 1.53  0.12; 1.41 | 0.23  0.20  0.20 | 0.00~2.02  0.00~3.58  0.00~2.12 | 0.09; 0.43  0.04; 1.04  0.13; 0.71 | 0.8659  0.8182  0.3813 |
| CD68^+^CD163^+^  Tumor (n=8 vs 19)  Stroma (n=12 vs 19)  Total (n=8 vs 19) | 20  57  60 | 0~118  0~245  0~154 | 2; 57  1; 163  2; 138 | 9  51  31 | 0~803  2~1753  1~1181 | 5; 35  17; 86  13; 42 | 0.8657  0.7118  0.7454 | 0.25  0.90  0.68 | 0.00~1.17  0.00~3.89  0.00~1.47 | 0.03; 0.78  0.02; 1.78  0.02; 1.38 | 0.15  0.52  0.30 | 0.00~7.82  0.02~19.71  0.01~12.16 | 0.09; 0.44  0.26; 0.95  0.15; 0.47 | 0.9895  0.9762  0.8255 |
| CD68^+^CD163^-^  Tumor (n=8 vs 19)  Stroma (n=12 vs 19)  Total (n=8 vs 19) | 139  108  255 | 7~365  0~1483  22~832 | 16; 265  38; 546  52; 472 | 44  168  130 | 1~1638  25~2493  9~1979 | 22; 57  59; 446  39; 161 | 0.3128  0.7646  0.3332 | 1.55  0.98  2.60 | 0.10~4.07  0.00~11.10  0.27~7.97 | 0.20; 3.26  0.61; 6.61  0.56; 5.40 | 0.57  1.98  1.29 | 0.01~15.97  0.29~28.02  0.12~20.36 | 0.34; 1.14  0.57; 3.51  0.49; 2.23 | 0.3274  0.9286  0.4251 |
| PD-L1^+^CD68^+^  Tumor (n=8 vs 19)  Stroma (n=12 vs 19)  Total (n=8 vs 19) | 4  3  6 | 0~370  0~271  0~302 | 0; 5  2; 21  1; 32 | 8  11  10 | 0~79  0~463  0~174 | 0; 18  3; 104  2; 57 | 0.3227  0.1415  0.5746 | 0.05  0.05  0.07 | 0.00~3.66  0.00~2.80  0.01~3.08 | 0.00; 0.08  0.01; 0.36  0.01; 0.29 | 0.09  0.13  0.09 | 0.00~0.77  0.00~4.03  0.00~2.18 | 0.00; 0.34  0.04; 1.15  0.03; 0.51 | 0.3917  0.1999  0.5407 |
| CD56bright  Tumor (n=8 vs 19)  Stroma (n=12 vs 19)  Total (n=8 vs 19) | 0  5  4 | 0~51  0~95  0~68 | 0; 9  1; 63  0; 38 | 1  19  9 | 0~61  0~209  0~71 | 0; 7  0; 51  0; 19 | 0.7257  0.8786  0.8413 | 0.00  0.07  0.04 | 0.00~0.54  0.00~3.86  0.00~0.70 | 0.00; 0.07  0.00; 0.77  0.00; 0.34 | 0.01  0.19  0.10 | 0.00~0.86  0.00~2.64  0.00~1.03 | 0.00; 0.09  0.00; 0.46  0.00; 0.34 | 0.4977  0.9595  0.7378 |
| CD56dim  Tumor (n=8 vs 19)  Stroma (n=12 vs 19)  Total (n=8 vs 19) | 9  19  12 | 0~169  1~1093  1~273 | 0; 98  5; 370  4; 212 | 5  39  25 | 0~414  0~944  0~415 | 0; 56  3; 314  1; 157 | 0.7727  0.7567  >0.9999 | 0.10  0.77  0.12 | 0.00~1.79  0.02~17.09  0.01~2.43 | 0.00; 1.31  0.06; 2.86  0.05; 1.86 | 0.11  0.50  0.28 | 0.00~5.82  0.00~8.99  0.00~6.16 | 0.01; 0.79  0.01; 4.42  0.01; 2.16 | 0.9686  0.6381  0.8184 |
| CD20^+^  Tumor (n=8 vs 19)  Stroma (n=12 vs 19)  Total (n=8 vs 19) | 36  339  337 | 16~526  19~2664  30~949 | 17; 401  63; 879  40; 429 | 16  128  49 | 2~549  9~1670  3~411 | 7; 53  28; 236  27; 159 | 0.1038  0.0543  0.0824 | 0.46  3.55  3.19 | 0.14~5.55  0.47~59.04  0.32~8.20 | 0.20; 3.05  0.66; 10.33  0.42; 4.45 | 0.22  1.22  0.63 | 0.03~4.66  0.11~5.94  0.04~3.64 | 0.14; 0.85  0.42; 2.16  0.39; 2.12 | 0.2220  **0.0254**  0.1063 |

Table S11 TLS in responders compared with non-responders in patients with adjuvant chemotherapy.

|  |  |  | (n=31) |  |  |  |  |  |  |  |  |  |  |  |
| --- | --- | --- | --- | --- | --- | --- | --- | --- | --- | --- | --- | --- | --- | --- |
|  | pCR | (n=4) |  | Non-pCR | (n=27) |  |  | MPR | (n=12) |  | Non-MPR | (n=19) |  |  |
| TLS | Median | Min~Max | 25%; 75%  percentile | Median | Min~Max | 25%; 75% percentile | P-value | Median | Min~Max | 25%; 75% percentile | Median | Min~Max | 25%; 75% percentile | P-value |
| counts/mm^2^ | 0.93 | 0.00~1.69 | 0.14; 1.59 | 0.14 | 0.00~5.29 | 0.00; 0.55 | 0.2919 | 0.95 | 0.00~3.50 | 0.24; 1.59 | 0.10 | 0.00~5.29 | 0.00; 0.24 | **0.0063** |
| μm^2^/mm^2^ | 82915 | 0~132469 | 11270; 129539 | 16244 | 0~589706 | 16244; 52768 | 0.4468 | 86759 | 0~589706 | 37828; 142605 | 12598 | 0~273583 | 0; 24245 | **0.0044** |

Abbreviations: TLS, tertiary lymphoid structures
